# Solution Blow Spinning of High-Performance Submicron Polyvinylidene Fluoride Fibres: Computational Fluid Mechanics Modelling and Experimental Results

**DOI:** 10.3390/polym12051140

**Published:** 2020-05-16

**Authors:** Rasheed Atif, Madeleine Combrinck, Jibran Khaliq, Ahmed H. Hassanin, Nader Shehata, Eman Elnabawy, Islam Shyha

**Affiliations:** 1 Department of Mechanical and Construction Engineering, Faculty of Engineering and Environment, Northumbria University, Newcastle upon Tyne NE1 8ST, UK; madeleine.combrinck@northumbria.ac.uk (M.C.); jibran.khaliq@northumbria.ac.uk (J.K.); islam.shyha@northumbria.ac.uk (I.S.); 2 Centre of Smart Nanotechnology and Photonics (CSNP), SmartCI Research Center, Alexandria University, Alexandria 21544, Egypt; ahassan@ncsu.edu (A.H.H.); nader83@vt.edu (N.S.); eman.elnabawy@smartci.alexu.edu.eg (E.E.); 3 Department of Textile Engineering, Faculty of Engineering, Alexandria University, Alexandria 21544, Egypt; 4 Department of Engineering Mathematics and Physics, Faculty of Engineering, Alexandria University, Alexandria 21544, Egypt; 5 USTAR Bioinnovations Centre, Faculty of Science, Utah State University, Logan, UT 84341, USA; 6 Kuwait College of Science and Technology (KCST), Doha District 13133, Kuwait

**Keywords:** CFD, SBS, nozzle, PVDF, fibres

## Abstract

Computational fluid dynamics (CFD) was used to investigate characteristics of high-speed air as it is expelled from a solution blow spinning (SBS) nozzle using a k-ε turbulence model. Air velocity, pressure, temperature, turbulent kinetic energy and density contours were generated and analysed in order to achieve an optimal attenuation force for fibre production. A bespoke convergent nozzle was used to produce polyvinylidene fluoride (PVDF) fibres at air pressures between 1 and 5 bar. The nozzle comprised of four parts: a polymer solution syringe holder, an air inlet, an air chamber, and a cap that covers the air chamber. A custom-built SBS setup was used to produce PVDF submicron fibres which were consequently analysed using scanning electron microscope (SEM) for their morphological features. Both theoretical and experimental observations showed that a higher air pressure (4 bar) is more suitable to achieve thin fibres of PVDF. However, fibre diameter increased at 5 bar and intertwined ropes of fibres were also observed.

## 1. Introduction

In 1969, polyvinylidene fluoride (PVDF) was first reported as thermoplastic polymer piezoelectric material (PEM) exhibiting the piezoelectric activity [1]. PVDF based PEMs are classified as stimuli responsive materials and have been employed as standalone or as matrices in composites and layered structures to fabricate stimuli responsive systems for applications such as drug delivery and tissue engineering [2,3,4]. One of the applications of PVDF based PEMs is intelligent clothing to sense user activities in sports and personalized health care [5,6,7]. As the precursor for textiles is yarn, which is produced from fibres, various fabrication methods have been employed to produce fibres, such as gas jet spinning, nozzle-free centrifugal spinning, rotary jet spinning, melt blow spinning and flash-spinning [8,9]. Out of these, electrospinning has been extensively used for the fabrication of fibres; however, it has some limitations. Firstly, it can only be used for polymer systems that are electrically conductive, and secondly, formation of a high fraction of β-phase (which has the highest piezoelectric response) is dependent on very high electric field making the process a safety hazard [10]. As there is electric field involved, it also requires the use of conductive collectors. It also has low yield making it a laborious process and unfit for scale-up demands.

SBS has emerged as an alternative technique to produce sub-micron/nano sized fibres and can relieve the user of the limitations posed by electrospinning. In SBS, a polymer is dissolved into a suitable solvent to reduce its viscosity as thin fibres cannot be produced with very viscous polymer melt [11,12]. The solution is then injected through a nozzle which is surrounded by a concentric outer pipe from which air is purged out. The solution interacts with the air and forms short fibres which fall on a collector. The advantage of SBS is that it can be applied to both electrically conducting and insulating systems and does not require the application of electric field and conductive collectors to initiate fibre processing [13,14]. Moreover, the yield of fibre production is about hundred times higher than that of electrospinning making it suitable for industrialisation [15,16]. The nozzle design is very critical in SBS as it significantly affects the airflow field distribution, the air velocity and morphology of the final product [17]. If the internal diameter of nozzle is too large, outsized droplets will be produced resulting in fibres with larger diameters. Similarly, a very small orifice will reduce the throughput, however, it has the potential of producing thin fibres.

The influence of the protrusion length of the polymer solution syringe on fibre dimensions was found to be insignificant [17]. Lou et al. [18] also reported that the effect of protrusion length has insignificant effect on the fibre morphology. They used four different protrusion lengths: 4, 2, 0 and −2 mm (minus sign shows that the syringe was retracted from the nozzle end by a distance of 2 mm). They reported that the air velocity reaches a maximum in the vicinity of 10–20 mm below the nozzle face. The maximum air velocities were in the range of 170–180 m/s. However, based on practical experiments, they reported that the retracted nozzles resulted in intermittent process with polymer solution blocking the nozzle end. The protruded syringe could produce fibres without such deficiencies.

The attenuation force in solution blow spinning (SBS) is pressurized air and various computational methods have been employed to numerically investigate the influence of air pressure and velocity on the fibre morphology [19,20,21]. The laminar flow model is considered as the simplest of all available models while the k-ε turbulence model is one of the most commonly used models in computational fluid dynamics (CFD) to simulate mean flow characteristics for turbulent flow conditions with more rapid convergence [18,22]. The k-ε turbulence model is effective for solving problems involving reverse flow [12,23]. It is a semi-empirical model based on model transport equations for the turbulence kinetic energy (*k*) and its dissipation rate (*ε*). Neglecting gravitational effects, the transport equations for the *k*-*ε* turbulence model are given below [18,24]:

(1) $$\frac{\partial \left(\rho k\right)}{\partial t}+\frac{\partial \left(\rho ku_{i}\right)}{\partial x_{i}}=\frac{\partial }{\partial x_{j}}\left[\left(\mu +\frac{\mu_{t}}{\sigma_{k}}\right)\frac{\partial k}{\partial x_{j}}\right]+2\mu_{t}\left(\frac{\partial u_{i}}{\partial x_{j}}+\frac{\partial u_{j}}{\partial x_{i}}\right)\frac{\partial u_{i}}{\partial x_{j}}-2\rho \varepsilon M_{t}^{2}$$

(2) $$\frac{\partial \left(\rho \varepsilon \right)}{\partial t}+\frac{\partial \left(\rho \varepsilon u_{i}\right)}{\partial x_{i}}=\frac{\partial }{\partial x_{j}}\left[\left(\mu +\frac{\mu_{t}}{\sigma_{\varepsilon }}\right)\frac{\partial \varepsilon }{\partial x_{j}}\right]+2C_{\varepsilon 1}\frac{\varepsilon }{k}\mu_{t}\left(\frac{\partial u_{i}}{\partial x_{j}}+\frac{\partial u_{j}}{\partial x_{i}}\right)\frac{\partial u_{i}}{\partial x_{j}}-C_{\varepsilon 2}\rho \varepsilon \left(\frac{\varepsilon }{k}+1\right)$$

where ρ is density kg/m^3^, k is turbulent kinetic energy m^2^/s^2^, *t* is time s, $u_{i}$ and $u_{j}$ are velocity fluctuations in the *i*th and *j*th directions, respectively, μ is viscosity kg/(m.s), $\mu_{t}$ is turbulent viscosity kg/(m.s), $\sigma_{k}$ and $\sigma_{\varepsilon }$ are turbulent Prandtl numbers for the kinetic energy and the dissipation rate, respectively, ε is dissipation rate of turbulent kinetic energy, $M_{t}$ is turbulent Mach number, $C_{\varepsilon 1}$ and $C_{\varepsilon 2}$ are parameters for *k-ε* turbulence model. The flow characteristics for solution blow spinning process have yet not been studied in detail. For example, when air is passed through the air inlet and moves towards the nozzle tip, whether the nozzle will get choked or not and what will be the influence of choking on the fibre morphology have yet not been reported. A nozzle is choked when the maximum mass flow rate has been reached [25]. Any additional increment in pressure will result in an increase in chamber pressure. Internally the pressure might increase to a value in excess of the rated mechanical strength of the nozzle material which will result in catastrophic failure of the device. Externally of the nozzle, an increase beyond choked conditions can lead to shock wave formation in the nozzle wake. If fluid coming out of the nozzle cannot expand isentropically due to choking an irreversible discontinuity arises called shockwave [26,27]. The shockwave is an abrupt disturbance that causes discontinuous and irreversible changes in fluid characteristics such as speed, density, temperature, and pressure. As a result of the gradient in temperature and velocity being caused by the shock, heat is transferred, and energy is dissipated within the gas. These processes are thermodynamically irreversible [28]. As the nozzle design and the attenuation force (pressurized air) are of utmost importance in SBS, both numerical (CFD) and experimental methods were used to investigate the fibre formation. CFD was used to investigate the convergence point for high speed air as it comes out of the nozzle. The polymer solution syringe was positioned such that it did not choke due to the reversal of air flow. The produced fibres did not show any bead formation at higher pressure values indicating that optimized SBS can successfully produce submicron PVDF fibres.

## 2. Experimental Work

### 2.1. Materials

PVDF (Kynar, melt viscosity: 23.0–29.0 MPa.s at room temperature) was supplied by ARKEMA (King of Prussia, PA, USA). N, N-Dimethyl Formamide (anhydrous, 98%) was purchased from Loba Chemie, Mumbai, India. Chemicals were used as received.

### 2.2. Nozzle Design

**Figure 1:**
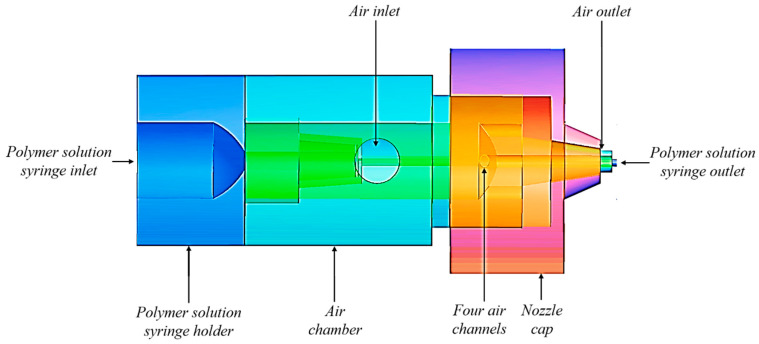
Different sections of solution blow spinning (SBS) nozzle.

**Figure 2:**
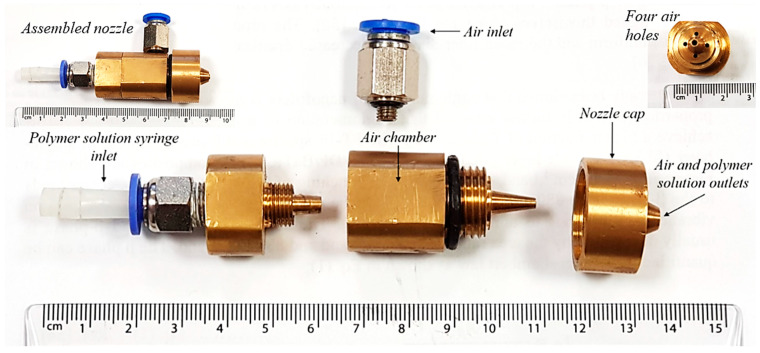
Various parts of the fabricated nozzle with four air holes through air chamber shown in the top right corner and assembled nozzle shown in the top left corner.

A bespoke SBS concentric nozzle was used to produce PVDF submicron fibres and was supplied by AREKA group, Istanbul, Turkey. A schematic of the nozzle used for the CFD model to predict air flow characteristics is shown in Figure 1. The polymer solution syringe is inserted through the opening at the left end of the nozzle that passes all the way through and comes out from the right end of the nozzle. In the middle is an air inlet (~4 mm diameter) that transfers air into the air chamber. To build pressure, the air chamber has four holes with internal diameters of ~1 mm each. These holes are covered with a cap that leaves a very narrow fissure for the air to come out 360° around the concentric polymer solution syringe. To ease manufacturing, the nozzle consists of four metallic sections (Figure 2) which are assembled to form the nozzle unit. Consequently, the experiments were carried out using this assembled nozzle. Most of the CFD models in the reviewed literature were carried out in 2D systems which were then extrapolated to 3D [18]. However, in this study, a 3D nozzle was employed which was also used in the experiments and using the same system for theoretical calculations will provide a more realistic comparison.

### 2.3. Experimental Setup

**Figure 3:**
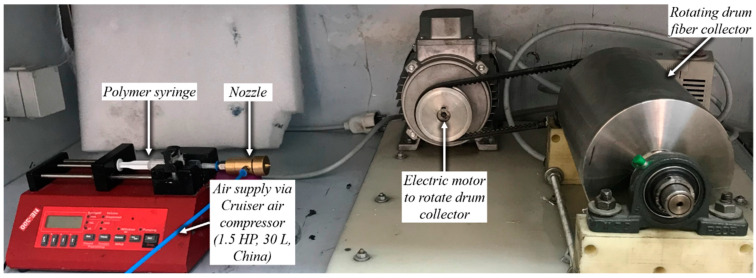
SBS setup for the production of polyvinylidene fluoride (PVDF) fibres.

**Figure 4:**
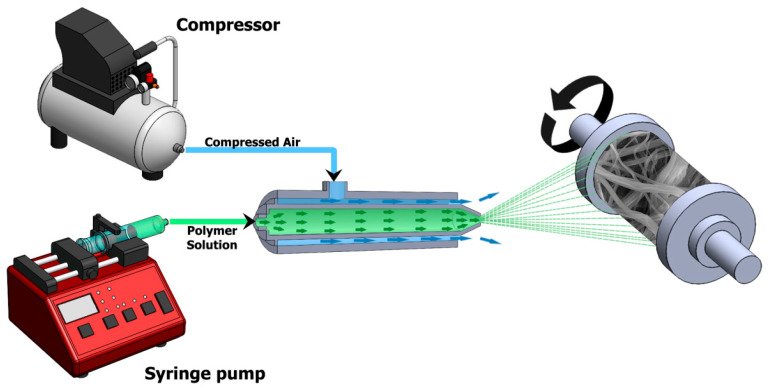
Schematic of solution blow spinning process.

The experimental setup is shown in Figure 3 and a schematic of the process is shown in Figure 4. As variation in temperature can influence fluid viscosity, and rheological properties are dependent on viscosity, it was ensured that the temperature of the polymer solution was held constant by keeping the SBS setup in a ventilated fume hood. No heating or cooling mechanism was used in the fume hood and the temperature during experiments remained in the range 22–25 °C. Pressurized air was used to attenuate polymer solution jet at 1–5 bar generated by Cruiser air compressor (1.5 HP, 30 L, Shanghai, China). NE-300 infusion syringe pump with 21-gauge needle was employed to feed the polymer solution, and was positioned inside the concentric nozzle with internal diameter di = 2 mm. A working distance of 20 cm was maintained between the nozzle and the drum collector.

### 2.4. Production of Samples/Fibres

A quantity of 15 wt % of PVDF polymer solution was pumped with the aid of the syringe and the feed rate of polymer solution was varied between 2 to 10 mL/h. Air pressure was varied between 1 to 5 bar (only one variable was changed at a time). The fibres were collected at the drum collector and analysed for their morphological features.

### 2.5. Characterization

CFD was carried out in ANSYS Fluent v19 (ANSYS Inc., Canonsburg, PA, USA). For the CFD simulation, a solid enclosure was built around the nozzle with zero shear slip and meshed consisting of 793,731 nodes and 3,355,960 elements. A steady-state, compressible Navier–Stokes equations was used whose component forms for continuity, x-momentum, y-momentum, z-momentum, and energy are given below, respectively, where *p* is pressure, q is heat flux, *u*, *v*, and *w* are velocity components, E_T_ is total energy, Re is Reynolds number, and Pr is Prandtl number.

(3) $$\frac{\partial \left(\rho u\right)}{\partial x}+\frac{\partial \left(\rho v\right)}{\partial y}+\frac{\partial \left(\rho w\right)}{\partial z}=0$$

(4) $$\frac{\partial \left(\rho u^{2}\right)}{\partial x}+\frac{\partial \left(\rho uv\right)}{\partial y}+\frac{\partial \left(\rho uw\right)}{\partial z}=-\frac{\partial p}{\partial x}+\frac{1}{Re}\left[\frac{\partial \tau_{xx}}{\partial x}+\frac{\partial \tau_{xy}}{\partial y}+\frac{\partial \tau_{xz}}{\partial z}\right]$$

(5) $$\frac{\partial \left(\rho uv\right)}{\partial x}+\frac{\partial \left(\rho v^{2}\right)}{\partial y}+\frac{\partial \left(\rho vw\right)}{\partial z}=-\frac{\partial p}{\partial y}+\frac{1}{Re}\left[\frac{\partial \tau_{xy}}{\partial x}+\frac{\partial \tau_{yy}}{\partial y}+\frac{\partial \tau_{yz}}{\partial z}\right]$$

(6) $$\frac{\partial \left(\rho uw\right)}{\partial x}+\frac{\partial \left(\rho vw\right)}{\partial y}+\frac{\partial \left(\rho w^{2}\right)}{\partial z}=-\frac{\partial p}{\partial z}+\frac{1}{Re}\left[\frac{\partial \tau_{xz}}{\partial x}+\frac{\partial \tau_{yz}}{\partial y}+\frac{\partial \tau_{zz}}{\partial z}\right]$$

(7) $$\begin{array}{ll} \frac{\partial \left(uE_{T}\right)}{\partial x}+\frac{\partial \left(vE_{T}\right)}{\partial y} & +\frac{\partial \left(wE_{T}\right)}{\partial z}\\ & =-\frac{\partial \left(up\right)}{\partial x}-\frac{\partial \left(vp\right)}{\partial y}-\frac{\partial \left(wp\right)}{\partial z}-\frac{1}{RePr}\left[\frac{\partial q_{x}}{\partial x}+\frac{\partial q_{y}}{\partial y}+\frac{\partial q_{z}}{\partial z}\right]\\ & +\frac{1}{Re}\left[\frac{\partial }{\partial x}\left(u\tau_{xx}+v\tau_{xy}+w\tau_{xz}\right)+\frac{\partial }{\partial y}(u\tau_{xy}+v\tau_{yy}+w\tau_{yz}\right)\\ & +\frac{\partial }{\partial z}\left(u\tau_{xz}+v\tau_{yz}+w\tau_{zz}\right)] \end{array}$$

The closure models used were the ideal gas model for density and the Sutherland equation for viscosity. The constants used were for air and mentioned below:

(8) μ=μ0(T0+CT+C)(TT0)32

where, *T*_0_ is reference temperature (K), µ_0_ is reference viscosity (1.716 × 10^−5^ kg/m.s) at the reference temperature *T*_0_ (273 K), *T* is effective temperature (110 K), and *C* is Sutherland’s constant for given gaseous material. The outlets were all specified as pressure far-field outlets. The inlet to the nozzle was a pressure inlet and remainder were all no-slip wall boundaries. Both laminar and k-ε turbulent simulations were carried out. Although the flow field is inherently turbulent, laminar flow was investigated as a steppingstone and was used to compare the results with turbulent flow simulation. The laminar results are not shown here but can be viewed in the Supplementary Materials (Figures S1–S22). A scanning electron microscope (JSM-6010LV-SEM, JEOL, Tokyo, Japan) was used to investigate the morphology of the produced fibres. Fibre diameter was analysed using Image-J software, average fibre diameter distribution was detected by measuring the distance across fibre boundaries at different imaging scales to ensure consistency.

## 3. Results and Discussion

**Figure 5:**
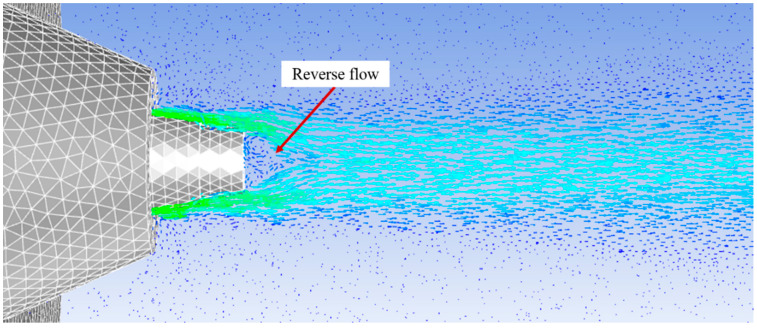
Reverse flow in the vicinity of the polymer solution syringe tip that can choke the syringe.

Under both laminar and k-ε turbulent flow conditions, a reverse flow was observed in the vicinity of polymer solution syringe outlet as shown in Figure 5. If a polymer solution droplet gets trapped by this reverse flow, there is high possibility that it will not move forward and will choke the syringe/nozzle. This supposition is further supported by the experimental observations reported by Lou et al. [18] who found that the retracted nozzle resulted in an intermittent process with polymer solution blocking the nozzle end. They also reported that the best morphology of fibres was produced when polymer syringe was protruded out by 4 mm.

Velocity, pressure, temperature, turbulent kinetic energy, and density contours at different air pressures are shown in Figure 6 and Figure 7. A magnified set of contours at 4 bar is shown in Figure 8 as the thinnest fibres were produced at that pressure. For clarity of readings large contours have been provided in the Supplementary Materials (Figures S23–S71). Velocity, temperature, and turbulent kinetic energy profiles at six vertical slices (at 0, 1, 4, 6, 16, and 26 mm from the nozzle end) were also calculated and plotted in Figure 9, Figure 10 and Figure 11, respectively. The values of the parameters and z-velocity were also measured at the horizontal symmetry axis of the geometry and are shown in Figure 12 and Figure 13. They will be referred to as centreline air velocity (*v_c_*), pressure (*P_c_*), temperature (*T_c_*), turbulent kinetic energy (*TKE_c_*), and density (*ρ_c_*) from hereafter.

At 1 bar, *v_c_* increases quadratically to ~80 m/s at a distance of ~0.7 mm from the air outlet, then slows down due to reverse flow until hits zero, and then shoots up to ~120 m/s at a distance of ~7 mm and then starts to gradually decrease. The exact value of air velocity may change due to polymer solution droplets in the flow field. Due to flow reversal, the *P_c_* initially decreases quadratically to ~−3 kPa at a distance of ~0.5 mm, after which it starts to increase until hits zero at a distance of ~1 mm, saturates at ~3 kPa at a distance of ~1.7 mm, and then gradually flattens out. The minimum *T_c_* recorded at ~4 mm was 291 K (18 °C) which was 7 K cooler than the ambient temperature which was set at 298 K (25 °C). The maximum TKE_C_ was ~1 kJ/kg recorded at ~1.3 mm from the air outlet. Experiments were conducted at 1 bar in order to produce fibres using the setup shown in Figure 3. Despite changing the feed rate between 2 mL/h to 10 mL/h, fibres could not be formed. This was ascribed to that the polymer solution droplets kept falling on the floor just ahead of the nozzle. Therefore, it is believed that air velocity of 120 m/s is not high enough to generate PVDF fibres. Similar trends were observed at 2–10 bars for CFD results; however, fibre morphology was significantly affected by air pressure.

Fibres were successfully produced at 2 bar whose SEM image is shown in Figure 14a and diameter distribution in Figure 14b. Fibres with wide distribution of diameters (between 100–900 nm) were observed and it is important to mention that some beads were also observed on these fibres. At 3 bar, relatively thinner fibres (than at 2 bar) as shown in Figure 14c were produced. The fibres were almost defect free with fibre diameters in the range of 140–700 nm (Figure 14d). At 4 bar, Much thin and bead-free fibres were produced as shown in Figure 14e. Most of the fibres were in 100–350 nm range (Figure 14f) and these were the thinnest fibres produced in the current work. The fibres produced at 5 bar were relatively thick as shown in Figure 14g. There were some intertwined fibre ropes observed at 5 bar and diameter of each rope was measured to get mean fibre diameter of ~1.5 µm (Figure 14h). At 5 bar, there is drop in temperature due to Joule-Thomson effect up to ~251 K (−22 °C). This cryogenic environment can produce residual stresses in the fibres that might have caused them turn and twist around each other while either during flight or after hitting the drum collector and resulted in interlocked fibre ropes. The turbulent kinetic energy (Figure 13c,d) continues to rise with increasing air pressure and velocity and this increased turbulence might be another factor in destabilizing the fibres in flight.

**Figure 12:**
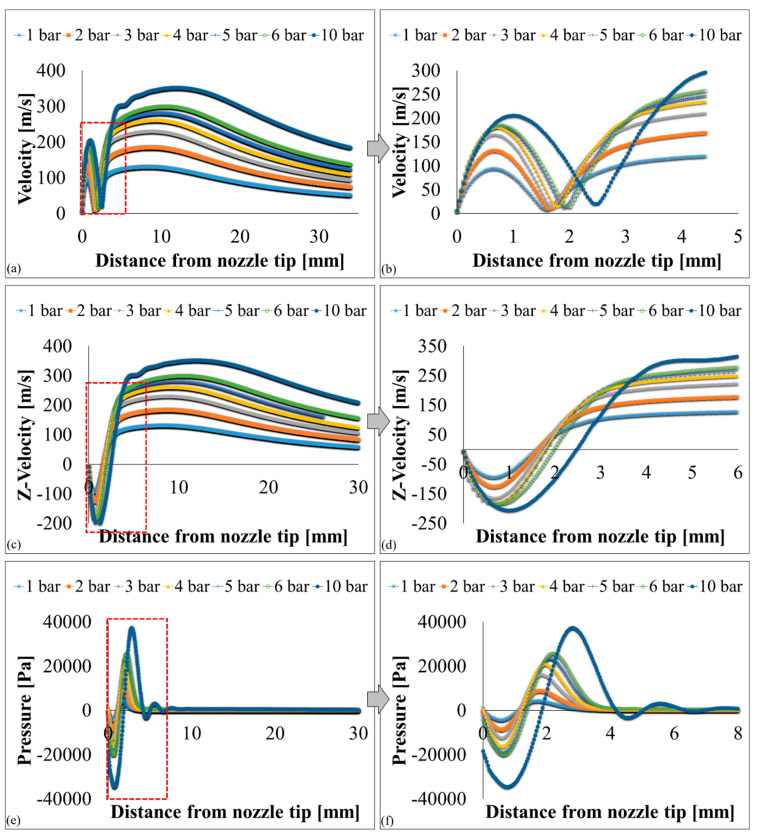
k-ε turbulence flow plots through SBS nozzle at different air pressures: (**a**,**b**) velocity (m/s), (**c**,**d**) z-velocity (m/s), and (**e**,**f**) pressure (Pa).

**Figure 13:**
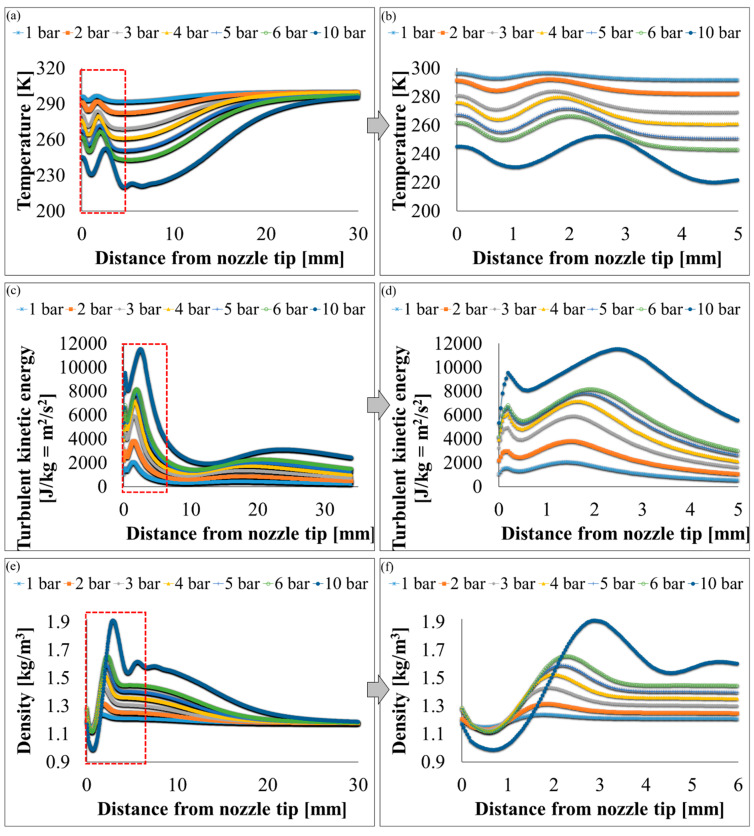
k-ε turbulence flow plots through SBS nozzle at different air pressures: (**a**,**b**) temperature (K), (**c**,**d**), turbulent kinetic energy (J/kg = m^2^/s^2^), and (**e**,**f**) density [kg/m^3^].

**Figure 14:**
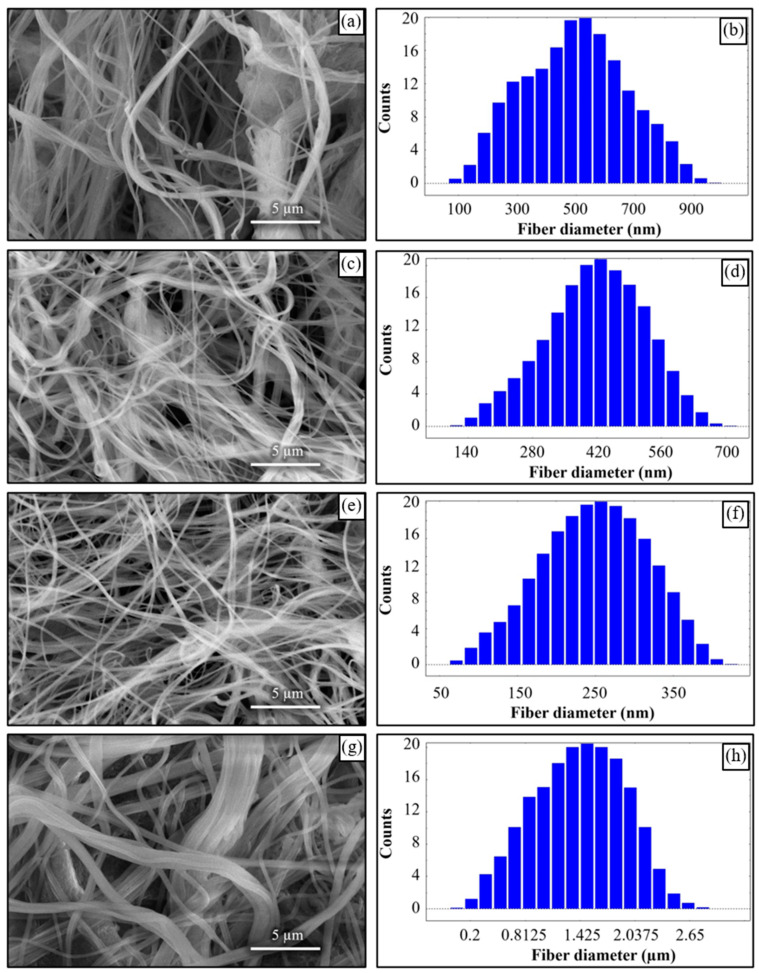
(**a**,**c**,**e**,**g**) SEM results at 2,3,4, and 5 bar air pressures, and (**b**,**d**,**f**,**h**) their respective fibre size distribution.

The z-velocity graphs (Figure 12c,d) hit negative values that confirm reverse flow. It is important to mention that the reverse flow is at the tip of syringe, which suggests that the polymer solution needs to be injected forcibly to pass the barrier created by the reverse flow (Figure 12e,f). There is fluctuation in the temperature as well (Figure 13a,b) due to reverse flow. As air leaves the air outlet, the temperature follows a sinusoidal curve dropping at a distance of ~1 mm from the air outlet, which goes up due to reverse flow, then drops again until it hits the lowest value (~193 K (−80 °C) at 10 bar) and finally gradually goes up to the room temperature. A similar trend was observed for turbulent kinetic energy (Figure 13c,d) along the positive ordinate.

The density plots (Figure 13e,f) show that the air is less dense at the nozzle opening. Air imposes frictional drag on the moving objects which is approximately proportional to the square of the velocity of the moving object. A decrease in density and viscosity would mean less frictional drag being exerted on the polymer solution droplet through the air flow field which would be able to move faster and will get thinner under the influence of attenuating force, i.e., high-speed air. The density graphs hit the peak values at ~4 mm from the nozzle end. This increment in density is propitious as it would exert compressive stresses on the fibres thereby resulting in compact fibres. 

A significant drop in temperature was observed as air comes out of the nozzle. It can possibly be related to the Joule–Thomson effect inducing temperature drops when high speed fluid quickly escapes through a narrow hole [29]. Static temperature drops during the isentropic expansion process and this drop in temperature is even more evident under supersonic flow where temperature can drop down to 213 K (−60 °C) without any cryogenic cooling or use of solid adsorption techniques [28]. It has been reported that PVDF solution temperature influences spinnability of PVDF fibres [15]. The drop in temperature can alter viscosity of the polymer solution as viscosity and temperature are inversely related to each other. When temperature of polymer solution is high, its viscosity will be low, and therefore low attenuation force will suffice to get thin fibres and vice versa. Attenuation force in SBS is high speed air which means that thin fibres can be achieved at relatively low air pressure and velocity. A higher air pressure results in a higher air velocity and turbulent fluctuations. The higher air velocity will result in thinner fibres but turbulent fluctuations may break the fibres [18]. It has been reported that there is a direct relationship between viscosity of polymer solution and mean fibre diameter. Haddadi et al. [30] incorporated hydrophobic and hydrophilic nanosilica into PVDF and reported that mean fibre diameter increased in both cases. They suggested that the viscosity of polymer solution increased by the incorporation of nanofillers which in turn led to an increase in mean fibre diameter. Yun et al. [31] fabricated Pb(Zr_0.53_Ti_0.47_)O_3_ reinforced PVDF nanofibres and reported that both density and viscosity of the polymer solution increased after the incorporation of PZT. The mean fibre diameter increased until 10 wt % and then gradually decreased when volume fraction was further increased up to 30 wt % [31].

The *v_c_* increases at 6 bar and 10 bar to ~400 and ~450 m/s, respectively. Experiments at 10 bar could not be performed due to the limitation of the setup. However, CFD results provide a useful information that the nozzle did not choke up until 10 bar. The effect of shock structures on the fibre formation has not been determined, however it is likely that the rapidly changing conditions before and after the shock will have a detrimental effect on the fibre morphology [32]. To avoid choking, nozzle diameter, feed rate and air pressure must be carefully optimized [32,33].

## 4. Conclusions

The current research provides a comprehensive numerical and experimental analysis for fibres produced using solution blow spinning (SBS). The flow characteristics of high-speed air through a bespoke nozzle were investigated numerically using computational fluid dynamics (CFD) and experimentally in a custom-built SBS setup to produce polyvinylidene fluoride (PVDF) submicron fibres. It has been concluded that under both laminar and k-ε turbulent flow conditions, a reverse flow was observed in the vicinity of polymer solution syringe outlet. As a result of this, if polymer solution droplets got trapped by this reverse flow, there is high possibility that the droplets will not move forward causing the syringe/nozzle to choke. However, chocking of nozzle does not take place at air pressures up to 10 bar. Submicron fibres were produced at air pressures ≥ 2 bar. However, higher pressure of 4 bar was found to be more effective in producing fibres in the range of 150–200 nm. Upon increasing the air pressure to 5 bar, the fibre diameter increased and interlaced fibre ropes were produced as significant temperature drop (~251 K (−22 °C)) was observed due to Joule–Thomson effect. The results presented in the paper will pave the way for future research in fibre manufacturing using SBS.

## Figures and Tables

**Figure 6:**
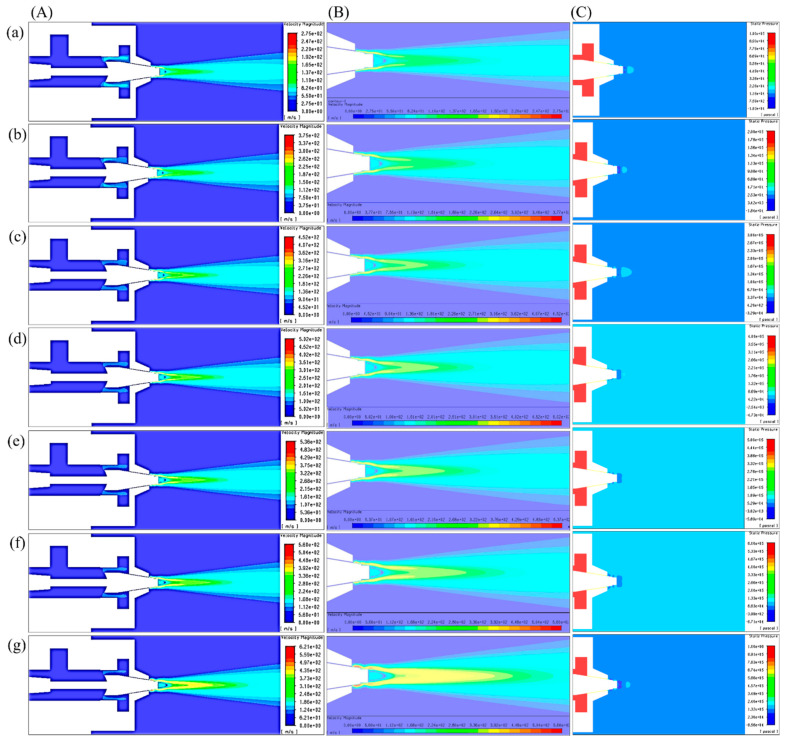
k-ε turbulence flow contours through SBS nozzle at different air pressures: (**A**,**B**) velocity (m/s), (**C**) pressure (Pa), (**a**) 1 bar, (**b**) 2 bar, (**c**) 3 bar, (**d**) 4 bar, (**e**) 5 bar, (**f**) 6 bar, (**g**) 10 bar.

**Figure 7:**
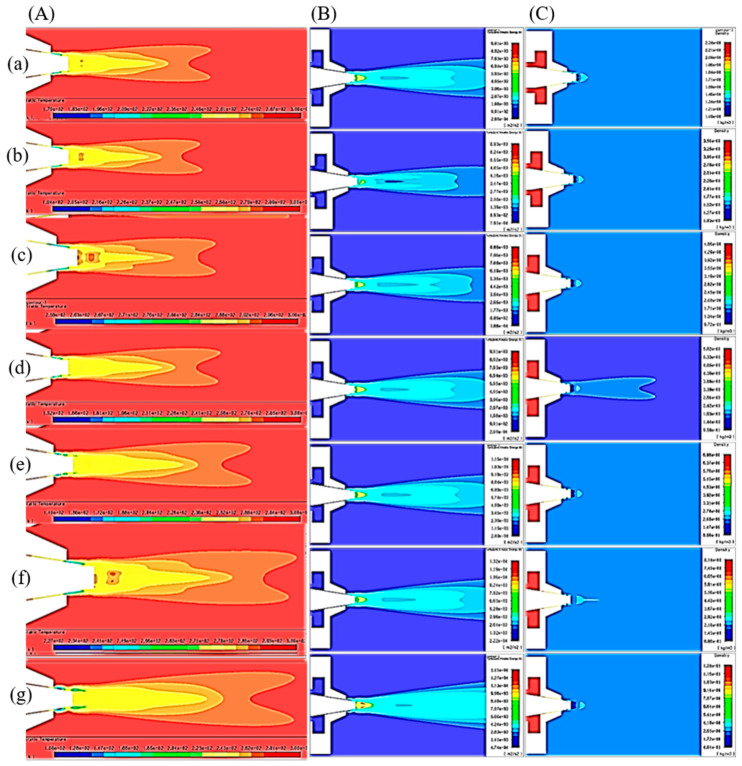
k-ε turbulence flow contours through SBS nozzle at different air pressures: (**A**) temperature (K), (**B**) turbulent kinetic energy (J/kg = m^2^/s^2^), and (**C**) density (kg/m^3^), (**a**) 1 bar, (**b**) 2 bar, (**c**) 3 bar, (**d**) 4 bar, (**e**) 5 bar, (**f**) 6 bar, (**g**) 10 bar.

**Figure 8:**
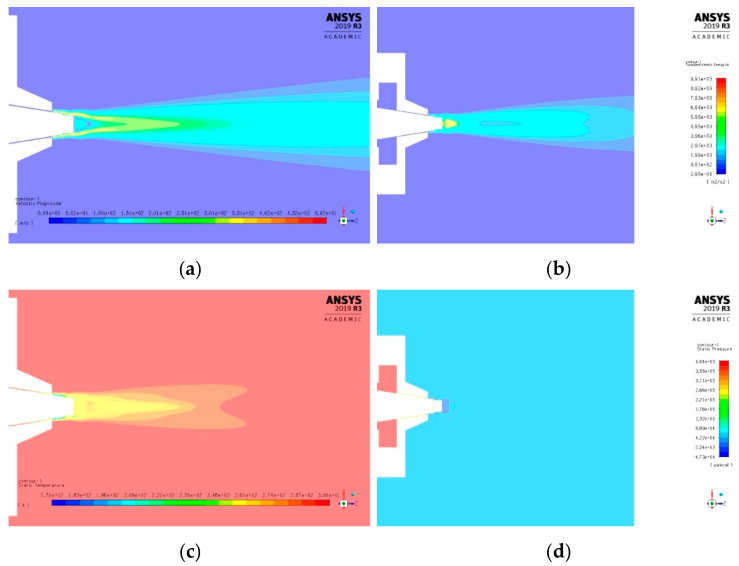
k-ε turbulence flow contours through SBS nozzle at air pressure of 4 bar: (**a**) velocity (m/s), (**b**) turbulent kinetic energy (J/kg = m^2^/s^2^), (**c**) temperature (K), and (**d**) pressure (Pa).

**Figure 9:**
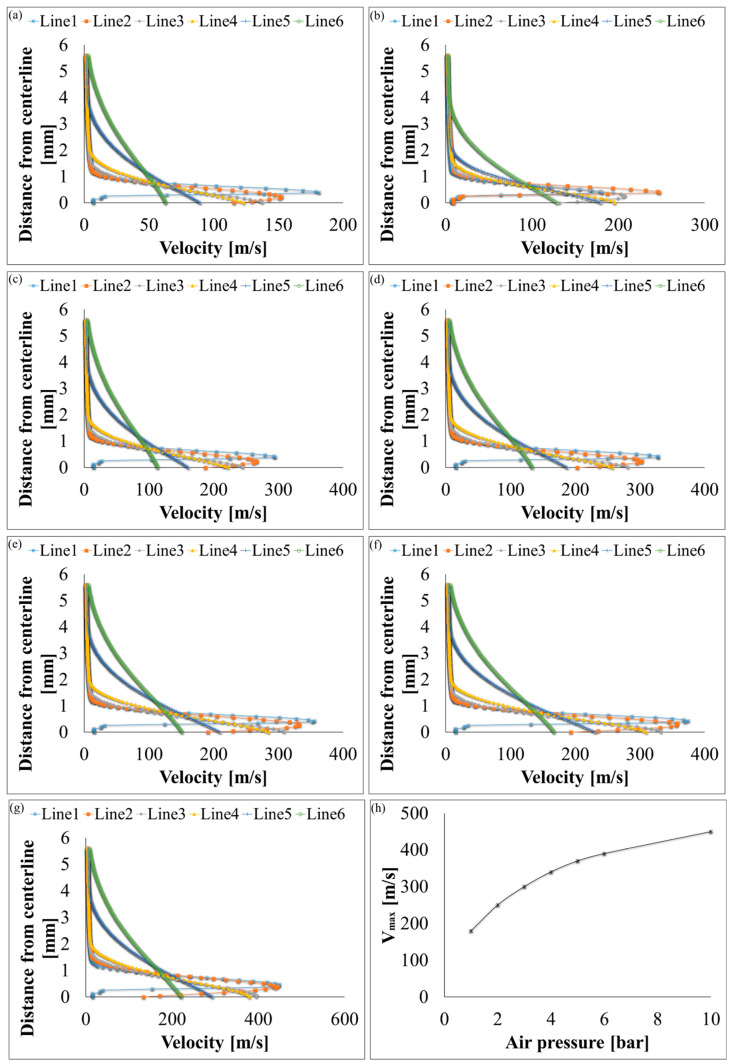
Velocity profiles at six vertical slices (at distance of 0, 1, 4, 6, 16, and 26 mm from the air outlet) for k-ε turbulence flow plots through SBS nozzle at different air pressures: (**a**) 1 bar, (**b**) 2 bar, (**c**) 3 bar, (**d**) 4 bar, (**e**) 5 bar, (**f**) 6 bar, (**g**) 10 bar, and (**h**) maximum velocity values at different air pressures.

**Figure 10:**
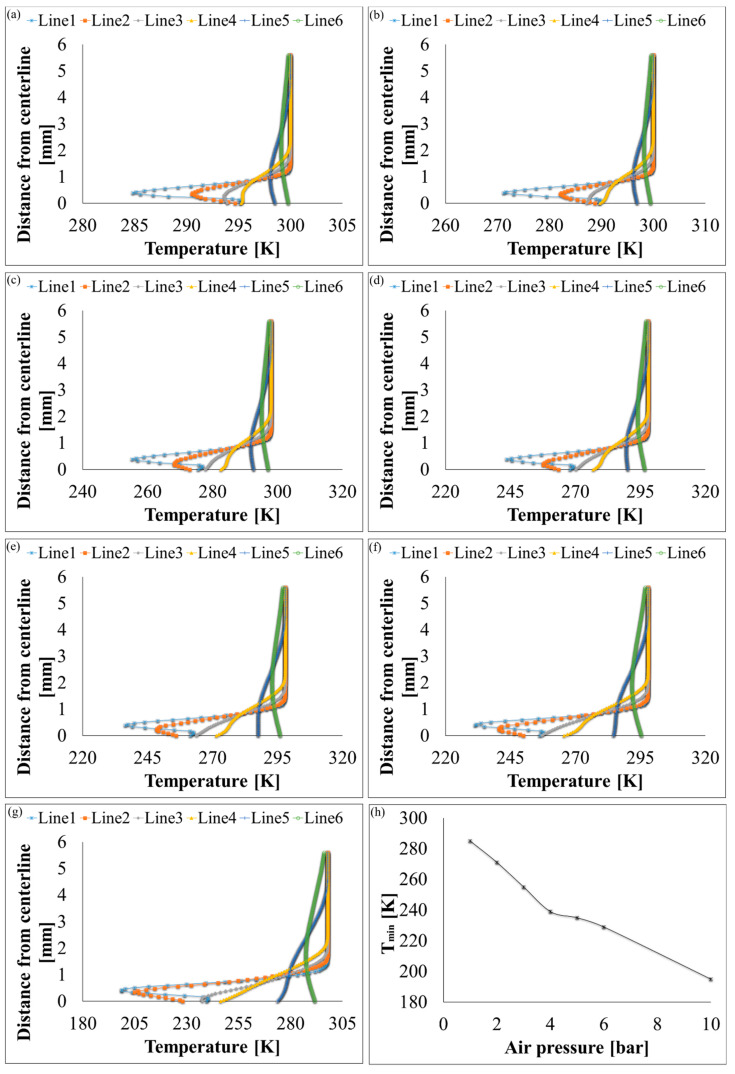
Temperature profiles at six vertical slices (at distance of 0, 1, 4, 6, 16, and 26 mm from the air outlet) for k-ε turbulence flow plots through SBS nozzle at different air pressures: (**a**) 1 bar, (**b**) 2 bar, (**c**) 3 bar, (**d**) 4 bar, (**e**) 5 bar, (**f**) 6 bar, (**g**) 10 bar, and (**h**) minimum temperature values at different air pressures.

**Figure 11:**
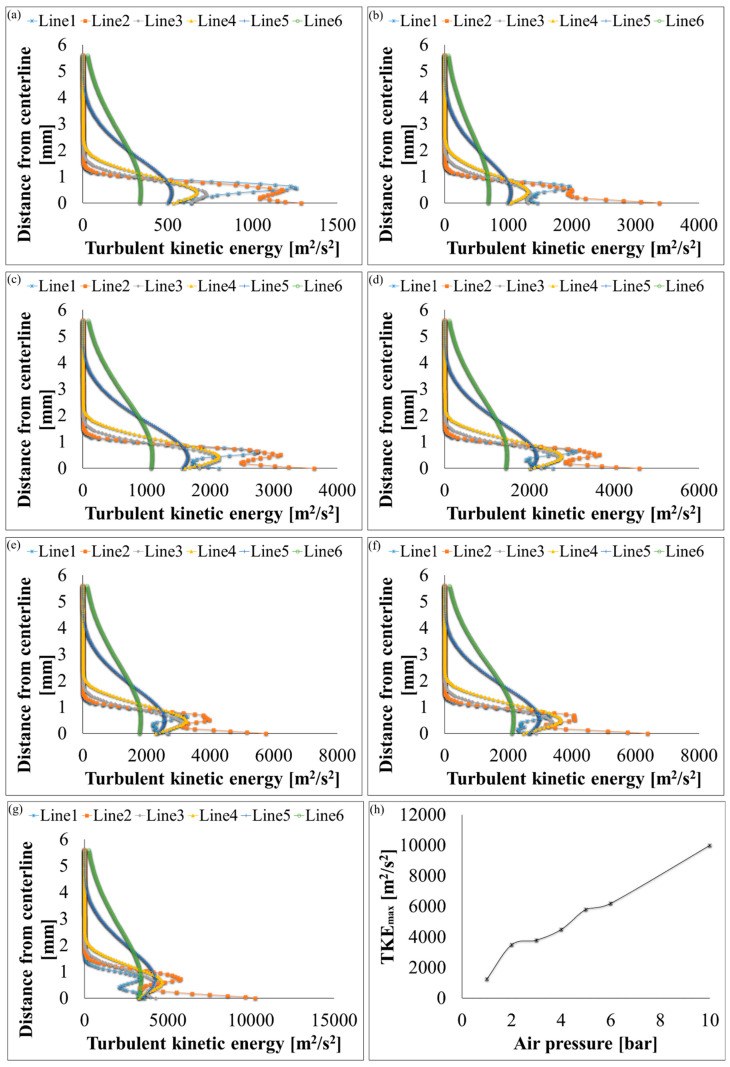
Turbulent kinetic energy profiles at six vertical slices (at distance of 0, 1, 4, 6, 16, and 26 mm from the air outlet) for k-ε turbulence flow plots through SBS nozzle at different air pressures: (**a**) 1 bar, (**b**) 2 bar, (**c**) 3 bar, (**d**) 4 bar, (**e**) 5 bar, (**f**) 6 bar, (**g**) 10 bar, and (**h**) maximum turbulent kinetic energy values at different air pressures.

## Supplementary Materials

The following are available online at https://www.mdpi.com/2073-4360/12/5/1140/s1, Figure S1. Solid enclosure with zero slip around SBS nozzle. Figure S2. Meshed system for CFD of SBS nozzle. The mesh comprises of 793,731 nodes with 33,55,960 elements. Figures S3–S12. Laminar flow results. Figures S13–S22. CFD contours for k-ε turbulent model at different values of *ε*_*c*1_ and *ε*_*c*2_. Figures S23–S71. k-ε turbulent model-based contours with default parameters.
