# Supplementary material for: Solution Blow Spinning of High-Performance Submicron Polyvinylidene Fluoride Fibres: Computational Fluid Mechanics Modelling and Experimental Results
Source: Polymers (Basel). 2020 May 16;12(5):1140. doi: 10.3390/polym12051140 (PMC7284647; doi:10.3390/polym12051140)
Supplement: Supplementary file 1 [file polymers-12-01140-s001.pdf]

polymers
MDPI

Supplementary Materials:

Solution Blow Spinning of High-Performance Submicron Polyvinylidene Fluoride Fibres: Computational Fluid Mechanics Modelling and Experimental Results

1. Laminar results:

For CFD, solid enclosure was built around the nozzle with zero shear slip as shown in Figure S1. The system was meshed consisting of 793,731 nodes and 3355,960 elements as shown in Figure S2. The velocity contour under laminar flow at 1 bar air pressure after 50 iterations is shown in Figure S3. The system is not fully converged but hints that the flow field is quite turbulent at the beginning as outcoming air has to push existing air outside the nozzle which scatters all around. At this stage feeding polymer solution is not recommended as any fibers produced will get scattered with air and will not be collected at the collector. The temperature contour is shown in Figure S4. It is interesting to note that temperature decreases in the vicinity of nozzle end. It can possibly be related to the Joule-Thomson effect which narrates that the temperature drops when high speed fluid quickly escapes through a narrow hole [1].

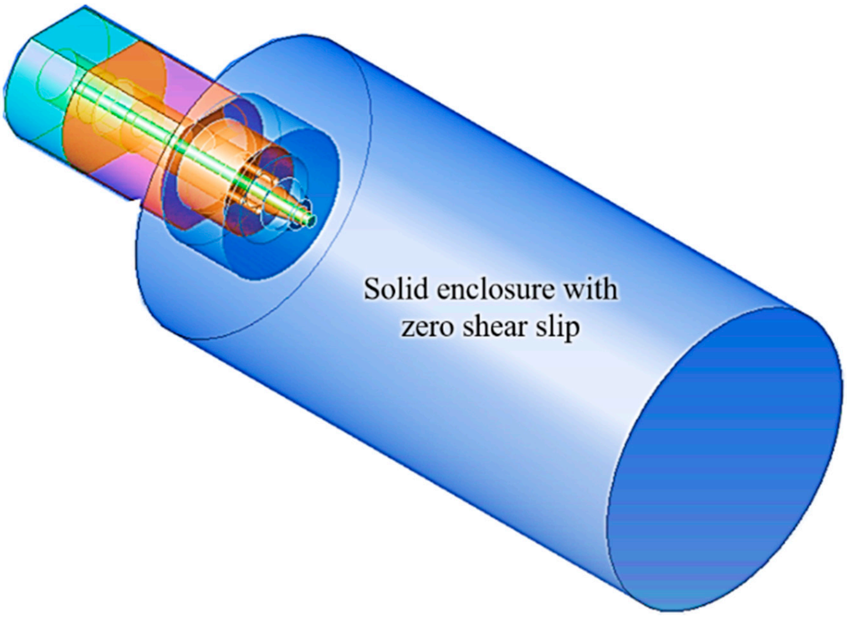

Figure S1. Solid enclosure with zero slip around SBS nozzle.

The density contour (Figure S5) shows that the air is less dense at the nozzle opening. A similar trend was observed for the molecular viscosity as shown in Figure S6. Air imposes frictional drag on the moving objects which is approximately proportional to the square of the velocity of the moving object. A decrease in density and viscosity would mean that it would exert less frictional drag on the polymer solution droplet through the air flow field which would be able to move faster and will get more thinner under the influence of attenuating force. A velocity contour of the fully converged system at 1 bar air pressure after 5000 iterations is shown in Figure S7. The velocity (~140 m/s) is maximum in the vicinity of air outlet and travels beeline until a distance of 35 mm from the air outlet after which it starts to scatter. The maximum air pressure outside the nozzle tip is around 2.15 × 10^4

Pa as shown in Figure S8. There is drop in temperature (up to ~12 °C) as shown in Figure S9. The flow fields were also investigated at 2 bar pressure and the velocity contour is shown in Figure S10. The air velocity increased to ~190 m/s and seems to be slightly more turbulent than at 1 bar beyond a distance of 35 mm from the air outlet. The maximum pressure in the vicinity of air outlet is 7.1×10^4 Pa as shown in Figure S11. A significant drop in temperature (~20 °C) was also observed at 2 bar as shown in Figure S12.

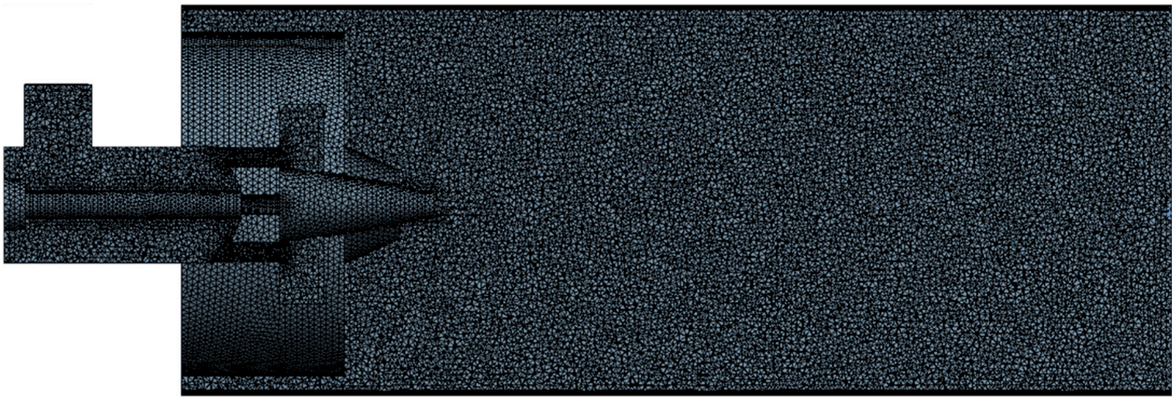

Figure S2. Meshed system for CFD of SBS nozzle. The mesh comprises of 793731 nodes with 3355960 elements.

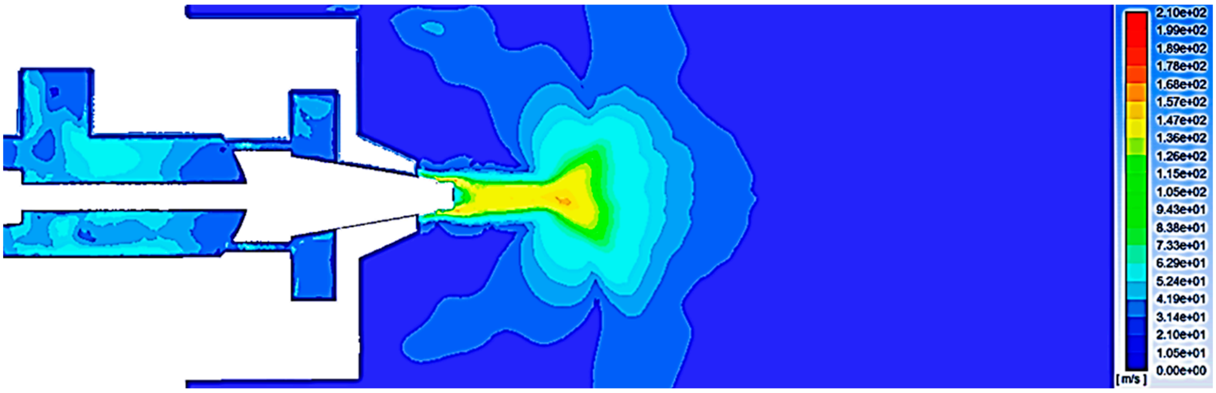

Figure S3. Velocity contour of laminar flow through SBS nozzle at 1 bar after 50 iterations.

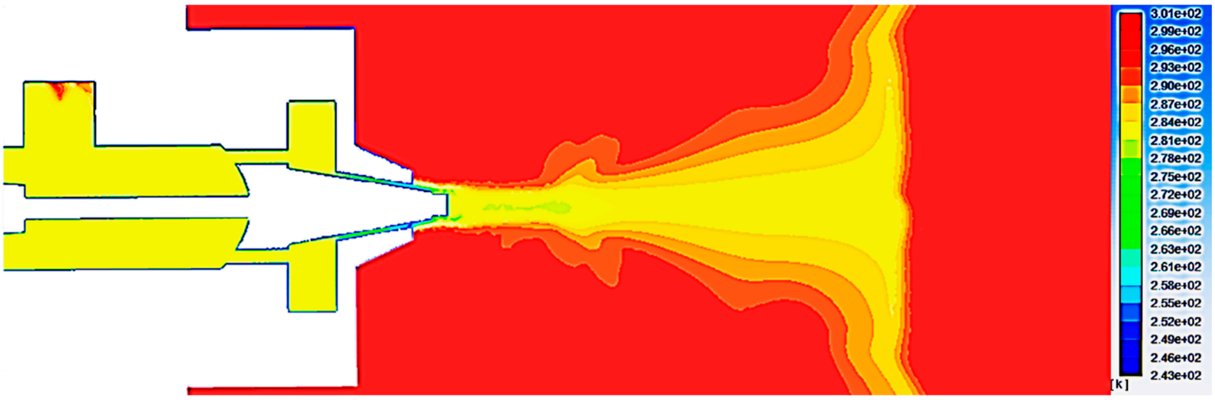

Figure S4. Temperature contour of laminar flow through SBS nozzle at 1 bar after 50 iterations.

2. Varying constants' values:

Under k-ε turbulence model, when ε_{C1} and ε_{C2} were set at 0.44 and 0.92, respectively, the resultant velocity contour is shown in Figure S13. The flow field is very scattered apart from the vicinity of the air outlet. Similar trend was observed in case of temperature as shown in Figure S14.

Contrary to laminar flow results (where drop in temperature was observed), temperature increased next to nozzle end. There are two regions of high temperature: one is right next to the nozzle end while other is at the far end of the fluid domain. These kinds of flow domains are unlikely to get in reality and suggest that low values of ε_{C1} and ε_{C2} yield erroneous results. To make a comparison, ε_{C1} and ε_{C2} values were significantly increased to 10.44 and 10.92, respectively. The resultant velocity contour is shown in Figure S15. The contour is much more confined and symmetric compared to what was achieved with ε_{C1} = 0.44 and ε_{C2} = 0.92. By using the default values of ε_{C1} and ε_{C2} of 2.44 and 2.92, respectively, the velocity contours with increasing iterations are shown in Figure S16– Figure S18. After 5000 iterations, much symmetric contour was observed. There is drop in temperature of ~8 °C (Figure S19) which is ~4 °C lower than what was achieved under laminar conditions. The velocity contour under k-ε turbulence model at 2 bar is shown in Figure S20. The air velocity is ~200 m/s and pressure (Figure S21) is ~4.5 × 10^4 Pa. There is increase in air velocity and pressure at 2 bar than at 1 bar. No shocks were generated as air velocity is in the subsonic regime. There is drop in temperature of ~17 °C as show in Figure S22.

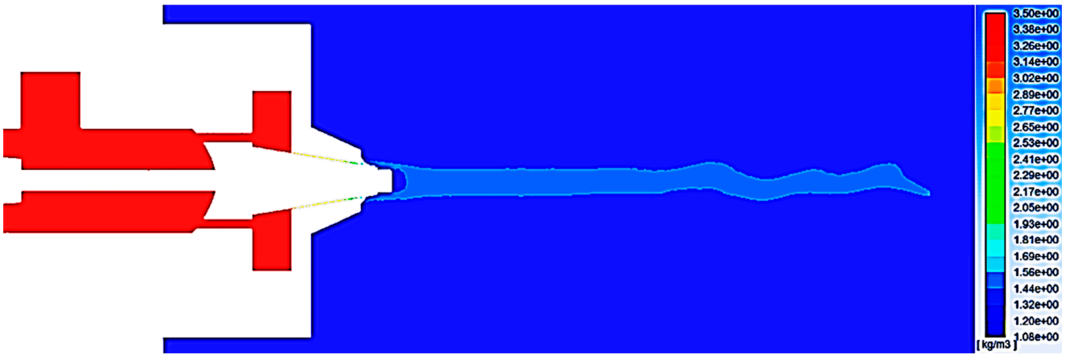

Figure S5. Density contour of laminar flow through SBS nozzle at 1 bar after 5000 iterations.

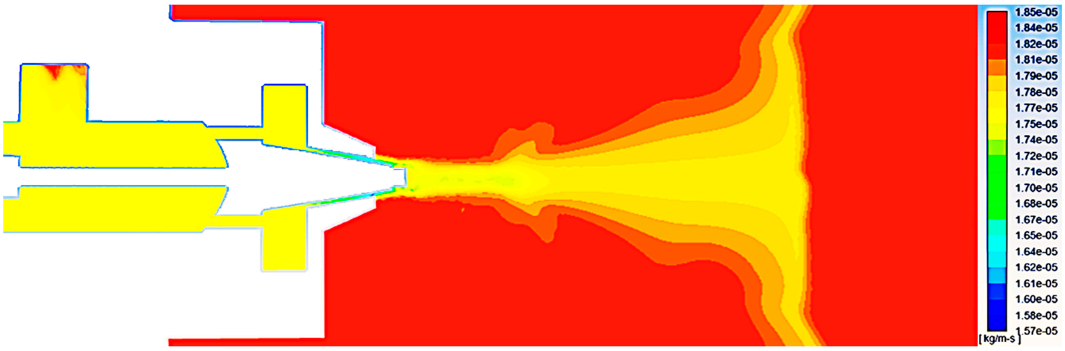

Figure S6. Molecular viscosity contour of laminar flow through SBS nozzle at 1 bar after 50 iterations.

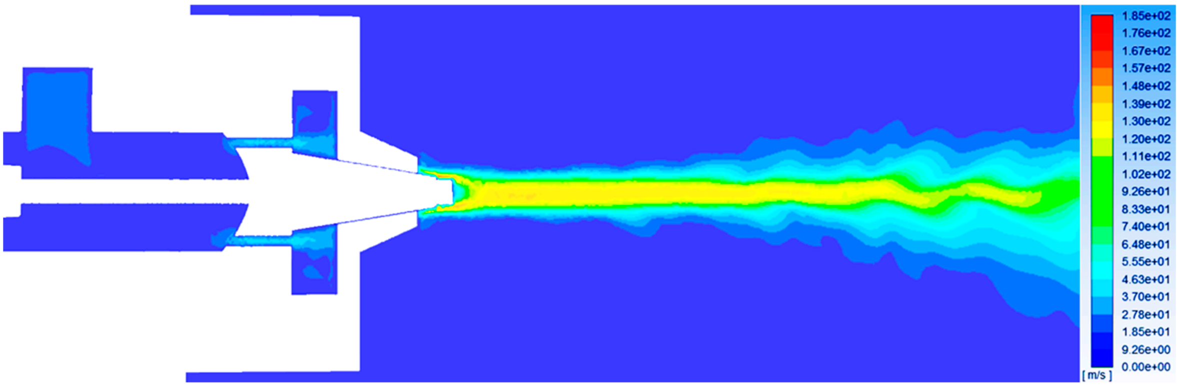

Figure S7. Velocity contour of laminar flow through SBS nozzle at 1 bar after 5000 iterations.

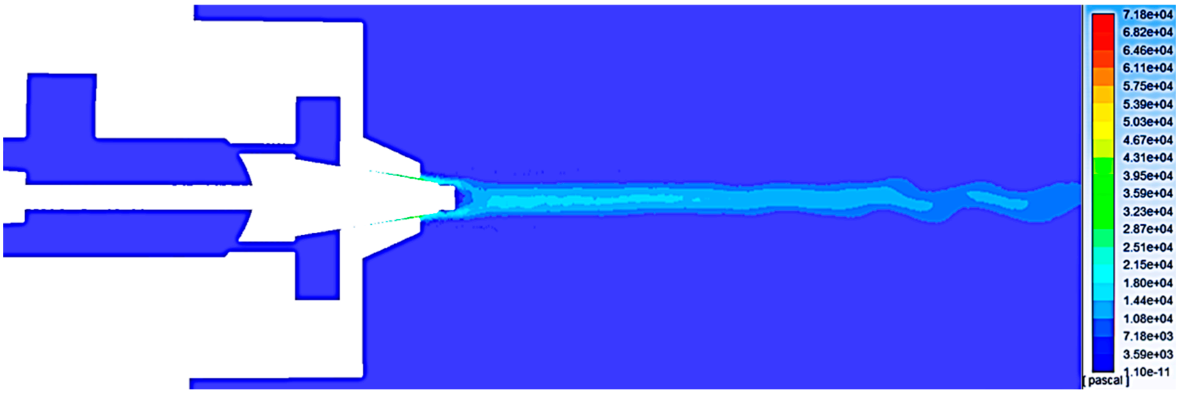

Figure S8. Pressure contour of laminar flow through SBS nozzle at 1 bar after 5000 iterations.

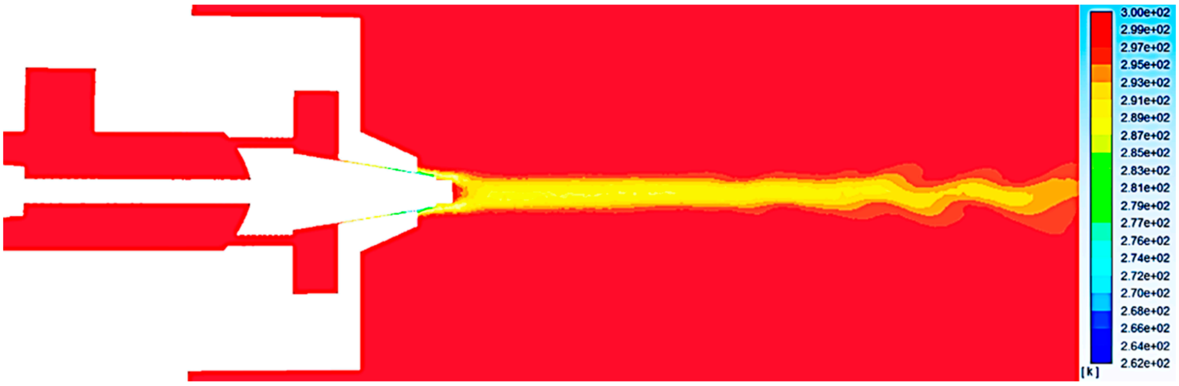

Figure S9. Temperature contour of laminar flow through SBS nozzle at 1 bar after 5000 iterations.

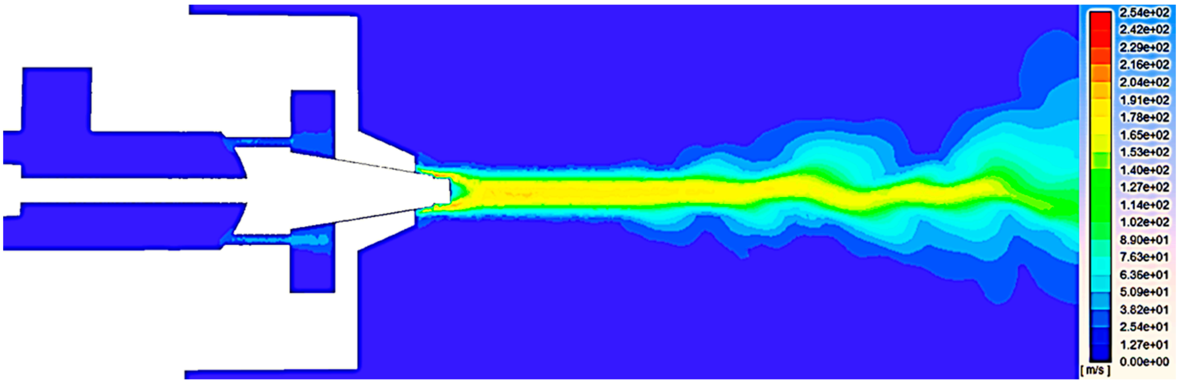

Figure S10. Velocity contour of laminar flow through SBS nozzle at 2 bar after 5000 iterations.

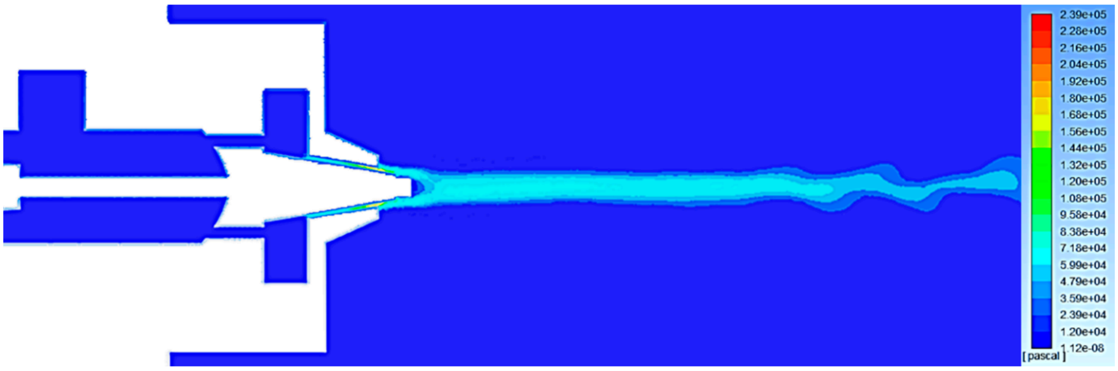

Figure S11. Pressure contour of laminar flow through SBS nozzle at 2 bar after 5000 iterations.

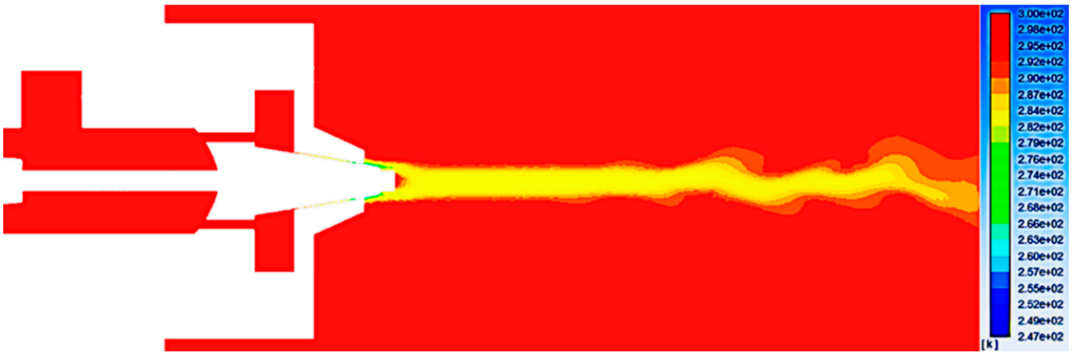

Figure S12. Temperature contour of laminar flow through SBS nozzle at 2 bar after 5000 iterations.

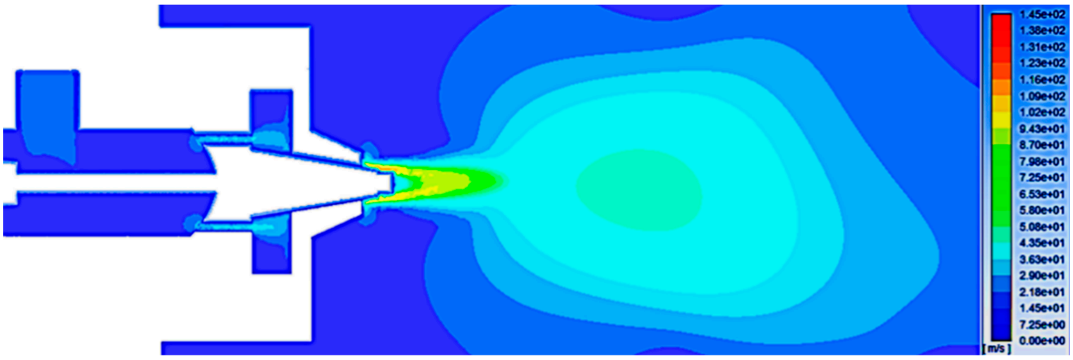

Figure S13. Velocity contour based on k-ε turbulence model with (εC1=0.44, εC2=0.92, Cmu=0.09) through SBS nozzle at 1 bar after 50 iterations.

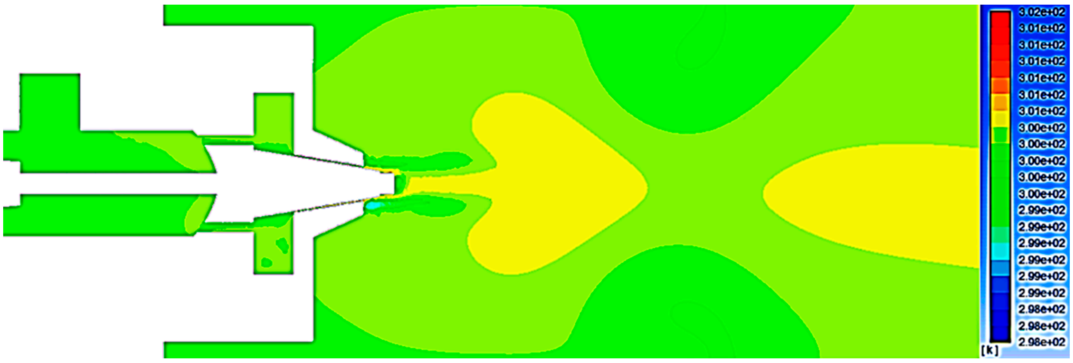

Figure S14. Temperature contour based on k-ε turbulence model with (εC1=0.44, εC2=0.92, Cmu=0.09) through SBS nozzle at 1 bar after 50 iterations.

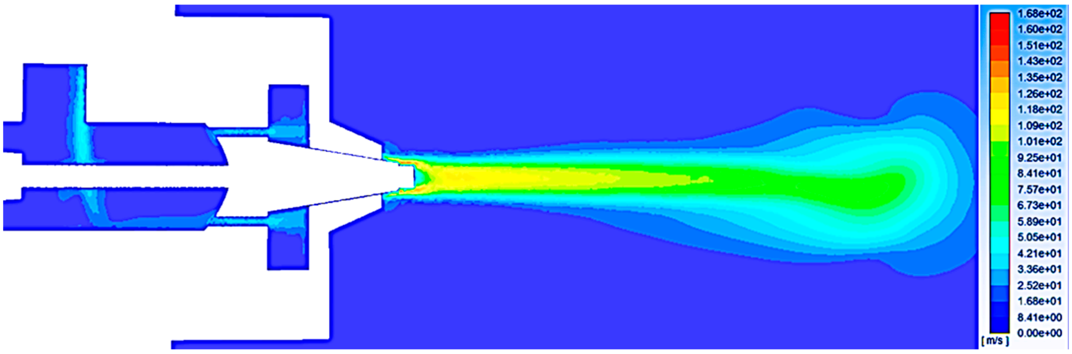

Figure S15. Residual chart based on k-ε turbulence model with (εC1=10.44, εC2=10.92, Cmu=0.09) through SBS nozzle at 1 bar after 1000 iterations.

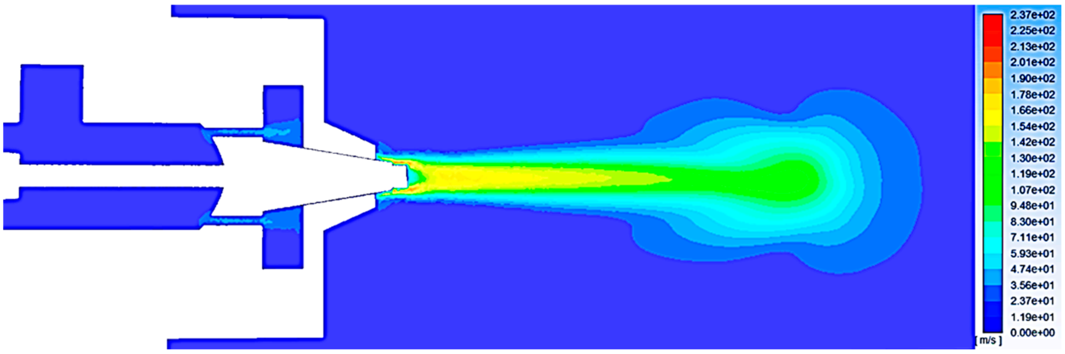

Figure S16. Velocity contour based on k-ε turbulence model with (εC1=2.44, εC2=2.92, Cmu=0.09) through SBS nozzle at 2 bar after 500 iterations.

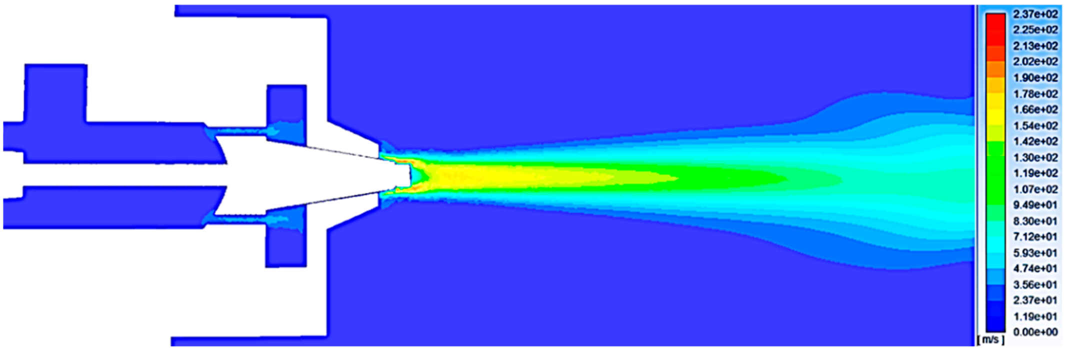

Figure S17. Velocity contour based on k-ε turbulence model with (εC1=2.44, εC2=2.92, Cmu=0.09) through SBS nozzle at 2 bar after 2000 iterations.

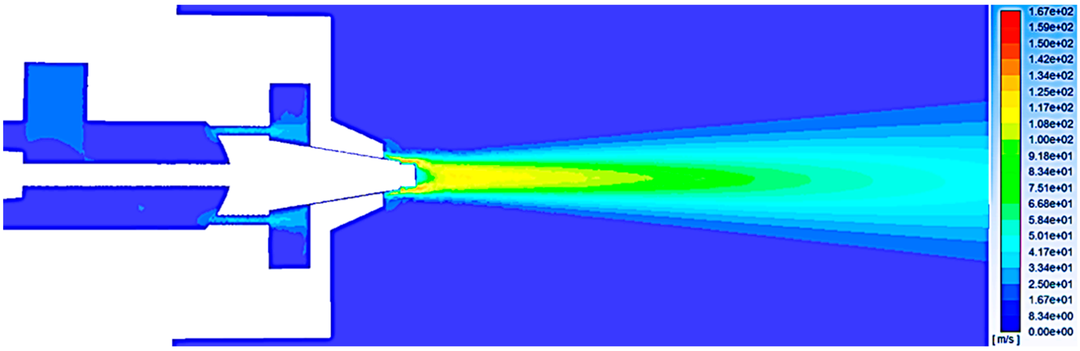

Figure S18. Velocity contour based on k-ε turbulence model with (εC1=2.44, εC2=2.92, Cmu=0.09) through SBS nozzle at 1 bar after 5000 iterations.

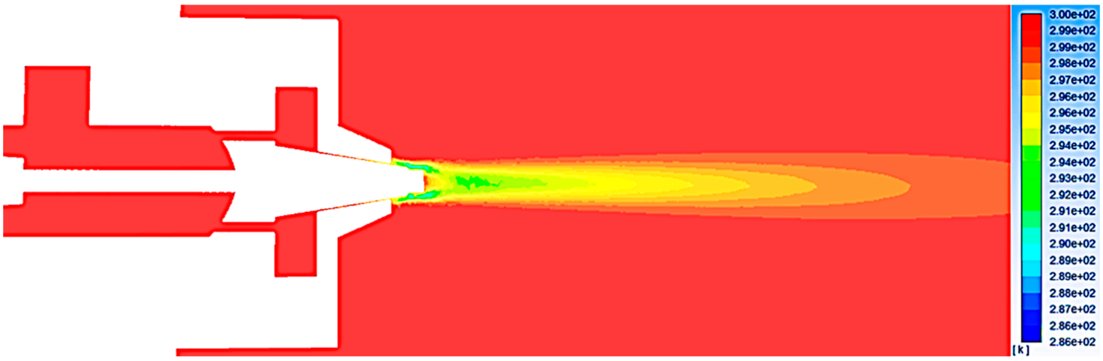

Figure S19. Temperature contour based on k-ε turbulence model with (εC1=2.44, εC2=2.92, Cmu=0.09) through SBS nozzle at 1 bar after 5000 iterations.

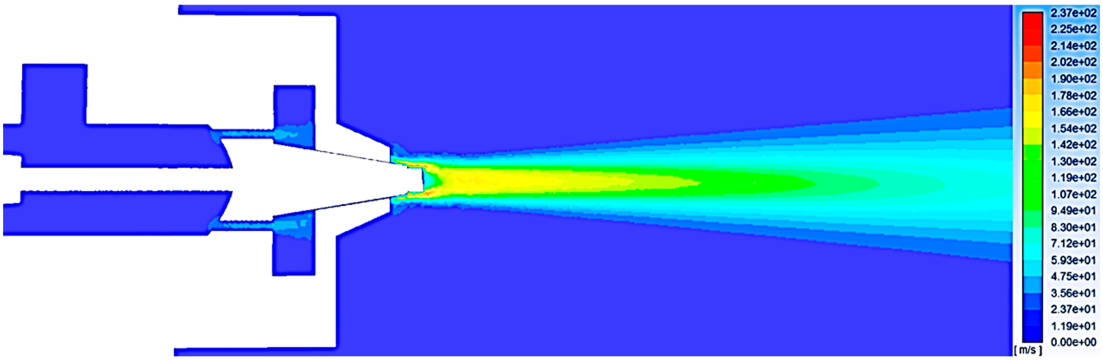

Figure S20. Velocity contour based on k-ε turbulence model with (εC1=2.44, εC2=2.92, Cmu=0.09) through SBS nozzle at 2 bar after 5000 iterations.

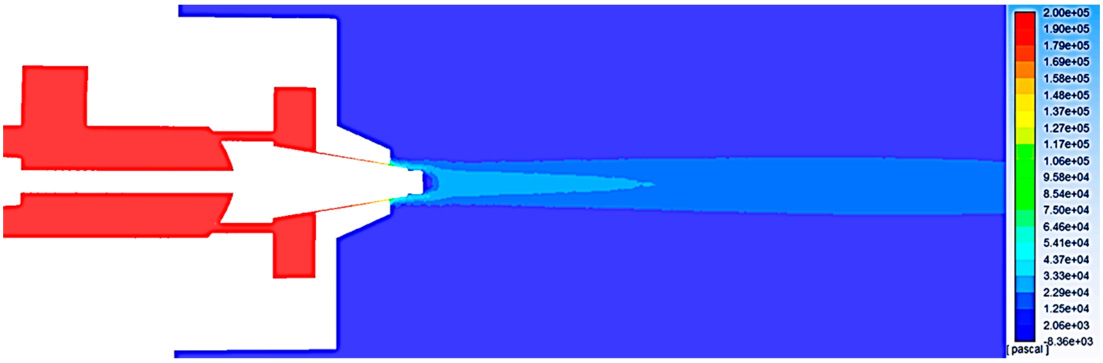

Figure S21. Pressure contour based on k-ε turbulence model with (εC1=2.44, εC2=2.92, Cmu=0.09) through SBS nozzle at 2 bar after 5000 iterations.

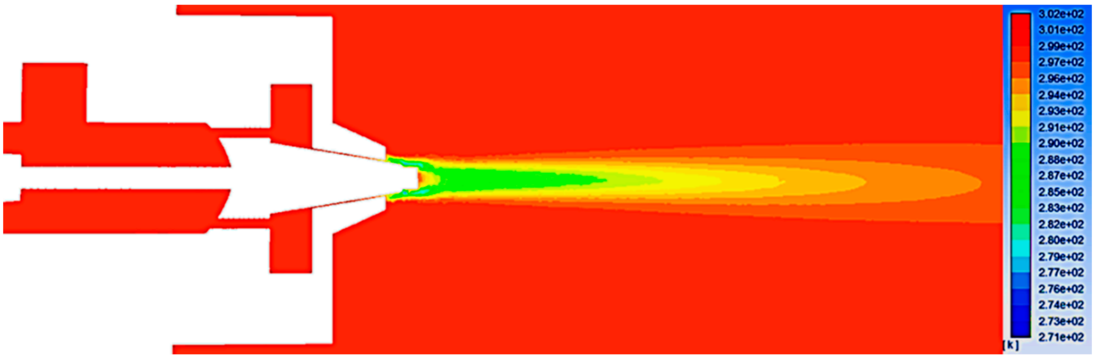

Figure S22. Temperature contour based on k-ε turbulence model with (εC1=2.44, εC2=2.92, Cmu=0.09) through SBS nozzle at 2 bar after 5000 iterations.

3. K-ε turbulence contours:

CFD contours for k-ε turbulent flow model at different pressures have been shown in Figure S23–S71. These results have been discussed in detail in the manuscript.

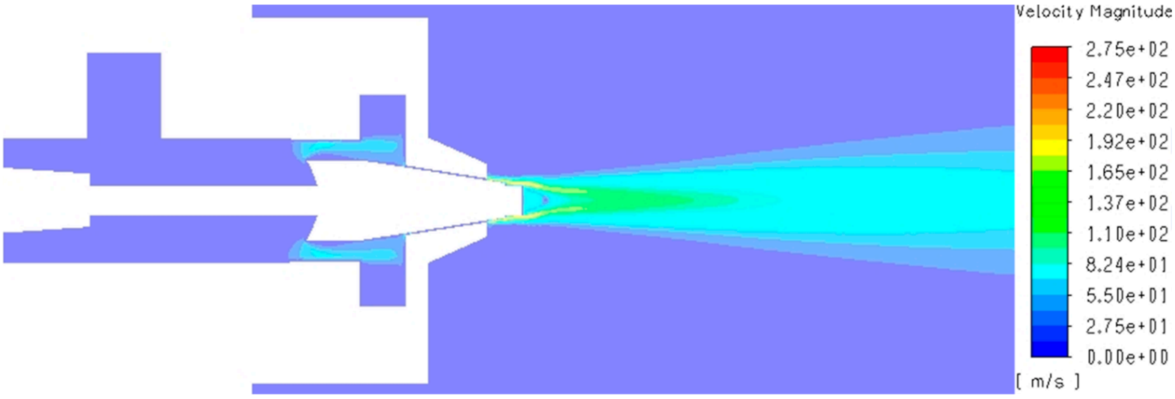

Figure S23. Velocity contour of k-ε turbulent flow through SBS nozzle at 1 bar.

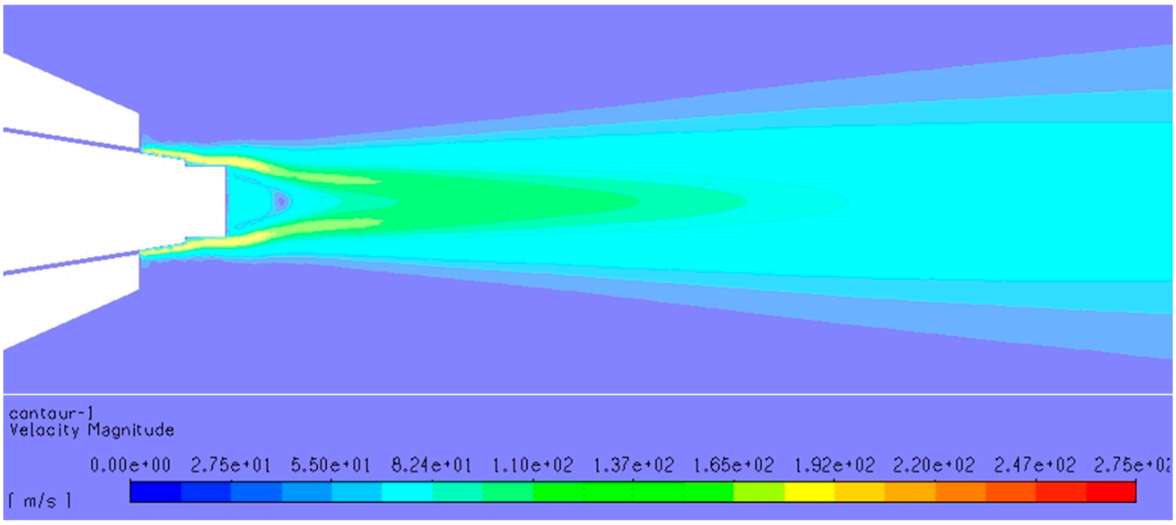

Figure S24. Velocity contour of k-ε turbulent flow through SBS nozzle at 1 bar.

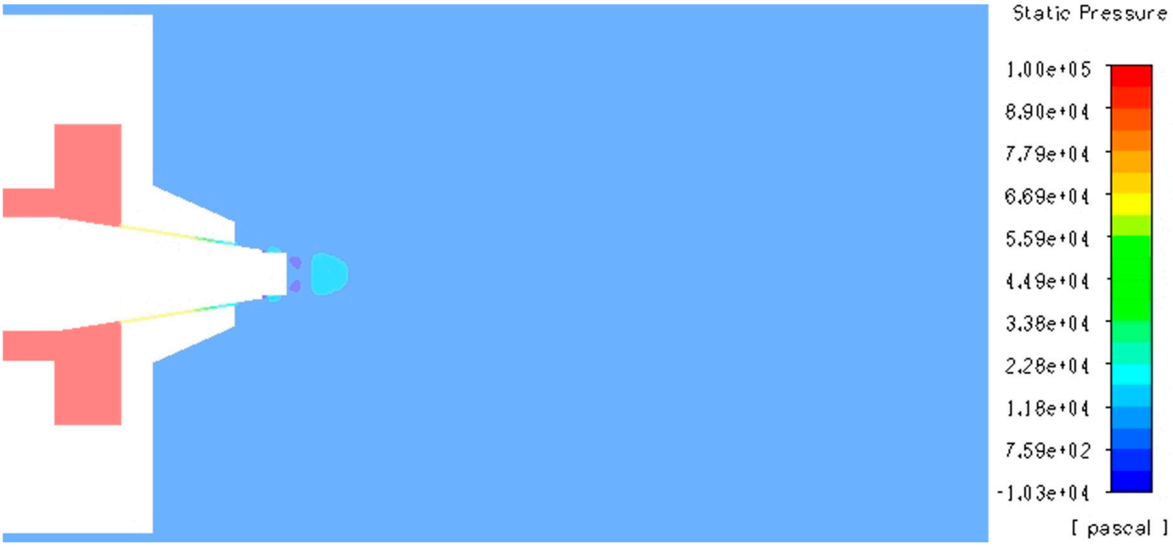

Figure S25. Pressure contour of k-ε turbulent flow through SBS nozzle at 1 bar.

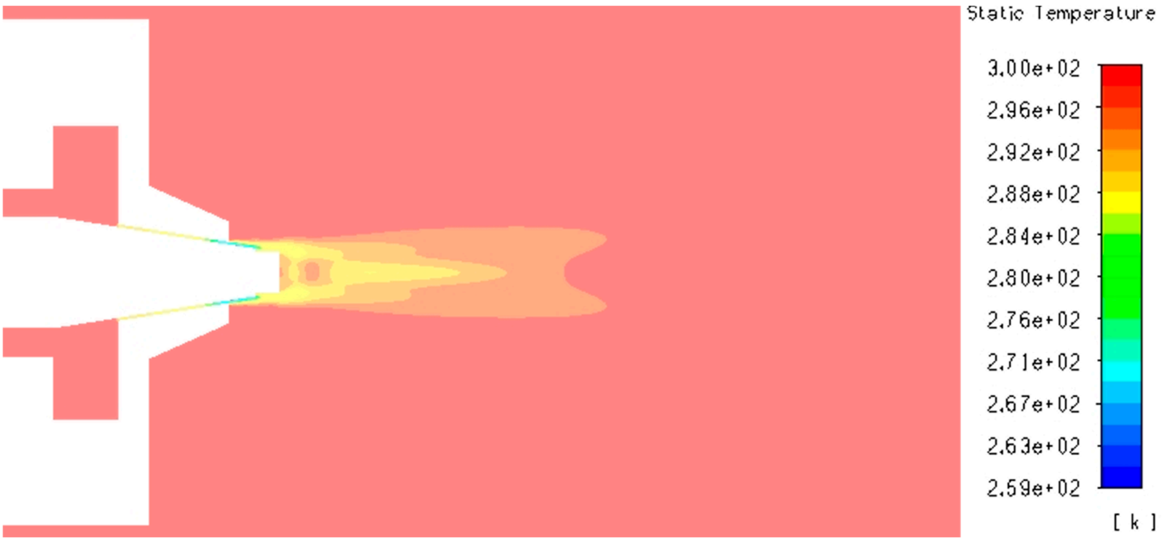

Figure S26. Temperature contour of k-ε turbulent flow through SBS nozzle at 1 bar.

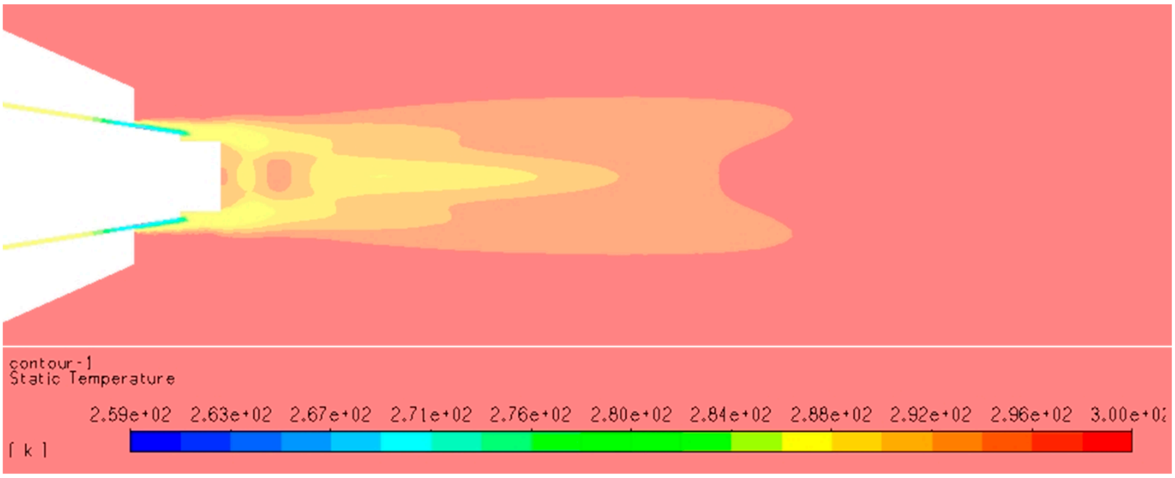

Figure S27. Temperature contour of k-ε turbulent flow through SBS nozzle at 1 bar.

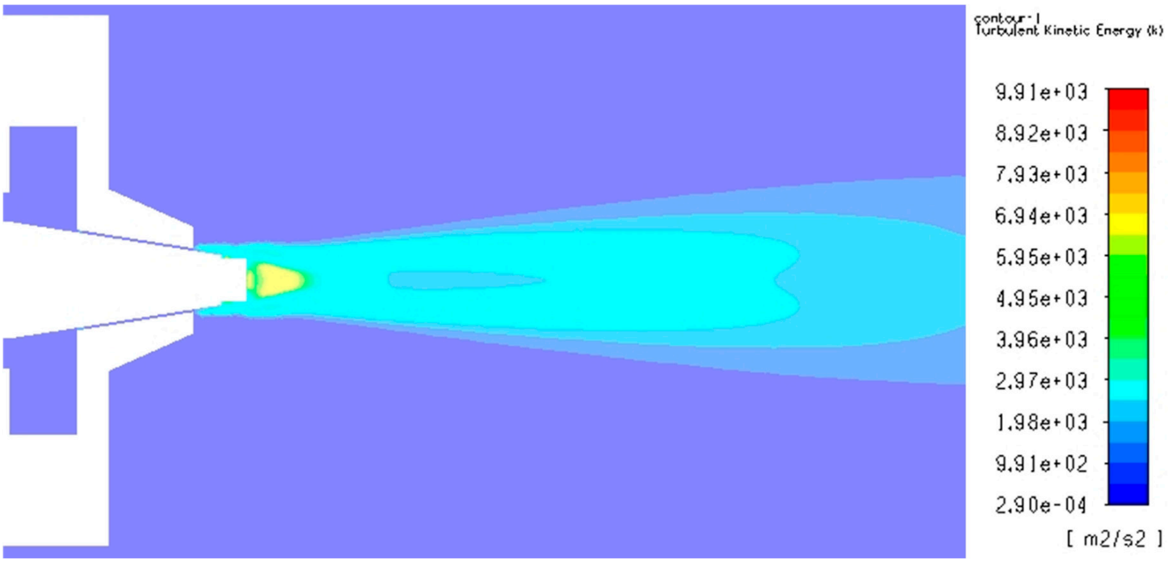

Figure S28. Turbulence contour of k-ε turbulent flow through SBS nozzle at 1 bar.

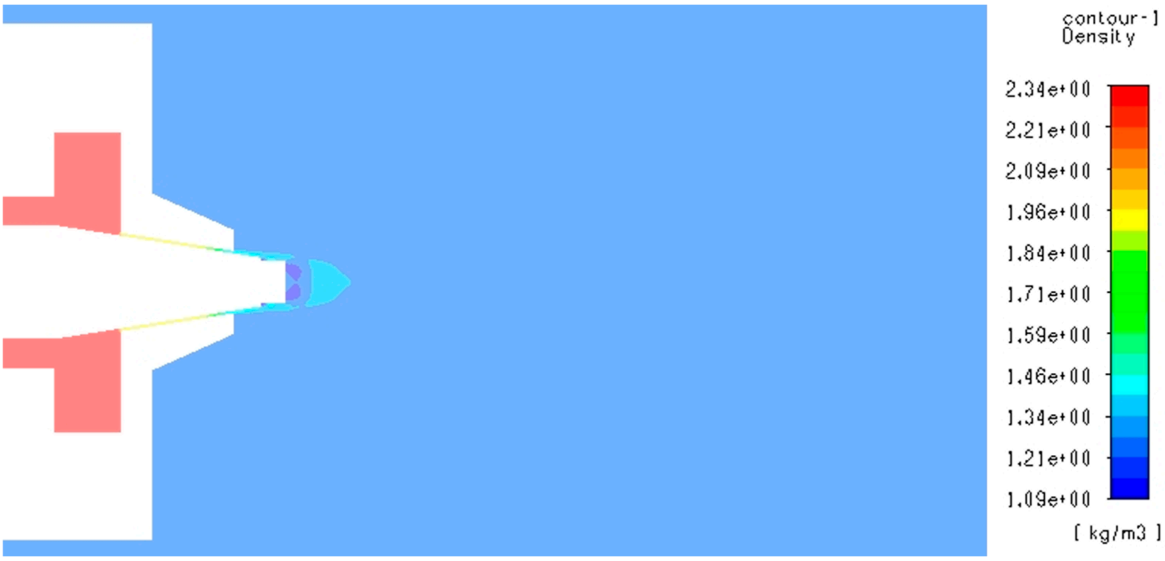

Figure S29. Density contour of k-ε turbulent flow through SBS nozzle at 1 bar.

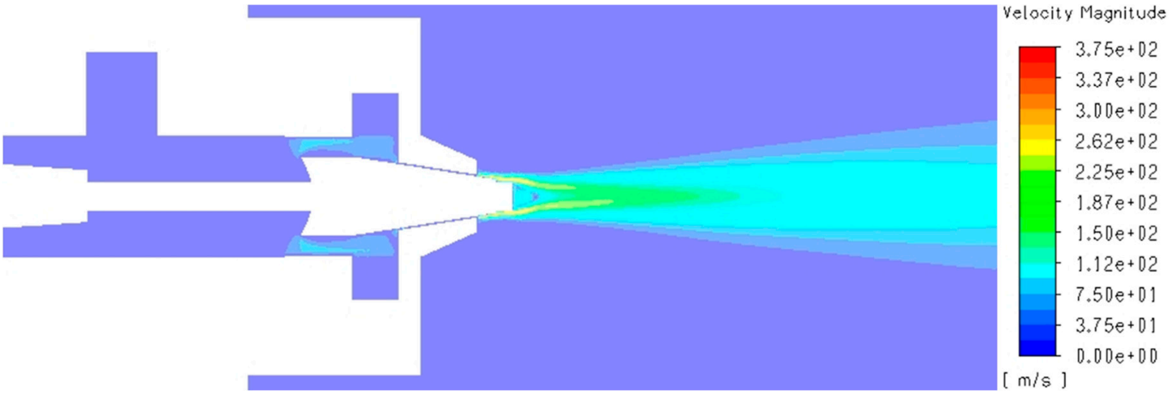

Figure S30. Velocity contour of k-ε turbulent flow through SBS nozzle at 2 bar.

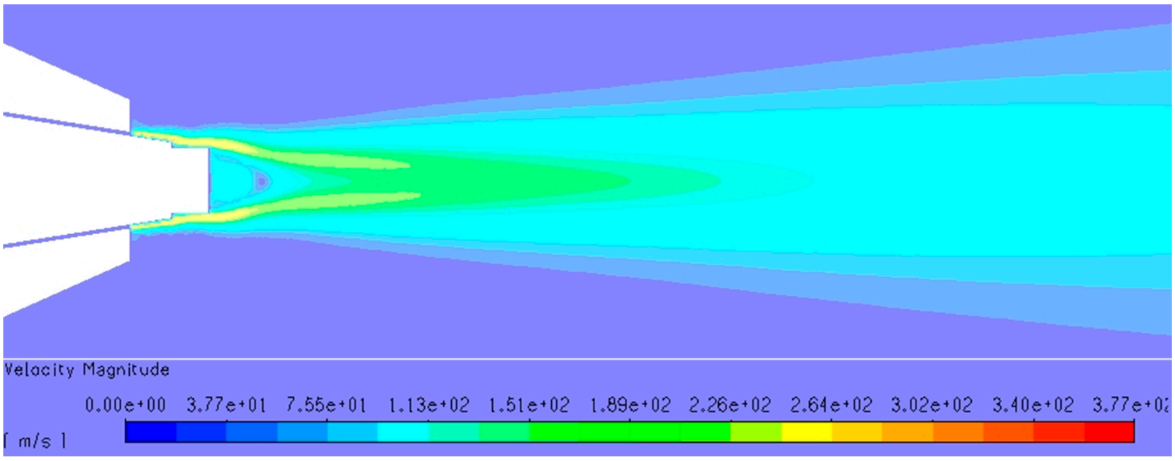

Figure S31. Velocity contour of k-ε turbulent flow through SBS nozzle at 2 bar.

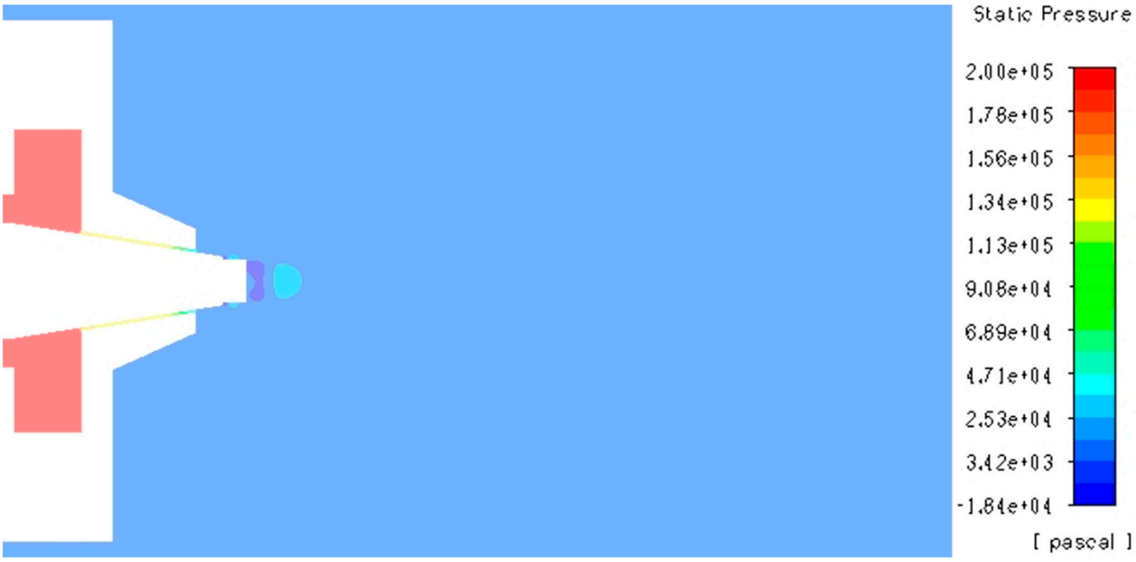

Figure S32. Pressure contour of k-ε turbulent flow through SBS nozzle at 2 bar.

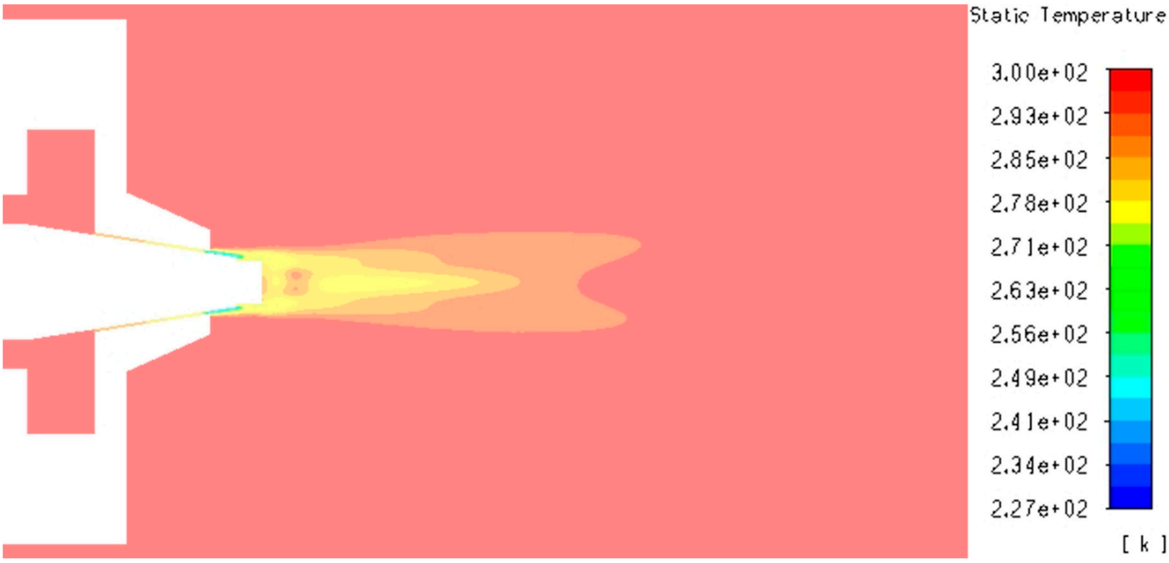

Figure S33. Temperature contour of k-ε turbulent flow through SBS nozzle at 2 bar.

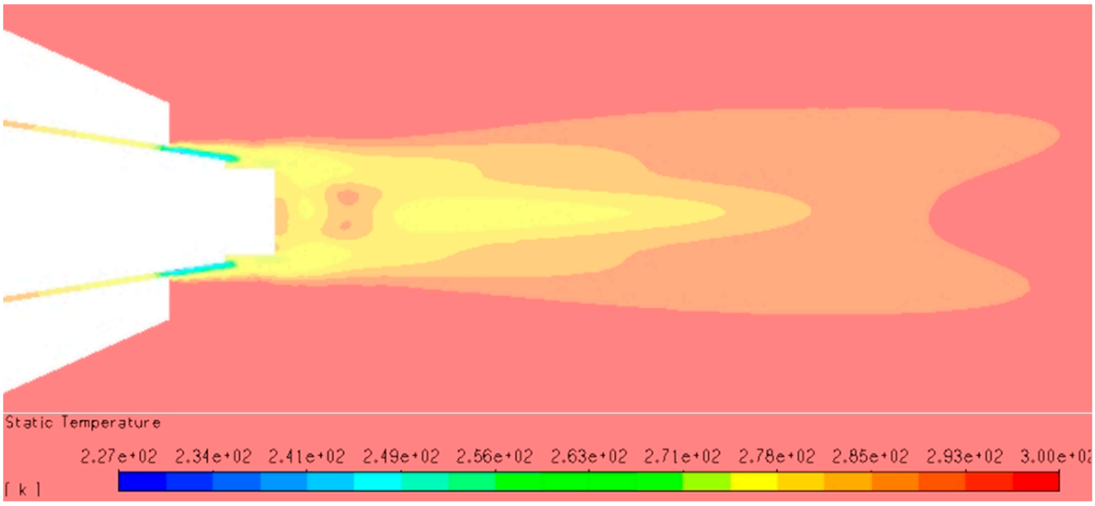

Figure S34. Temperature contour of k-ε turbulent flow through SBS nozzle at 2 bar.

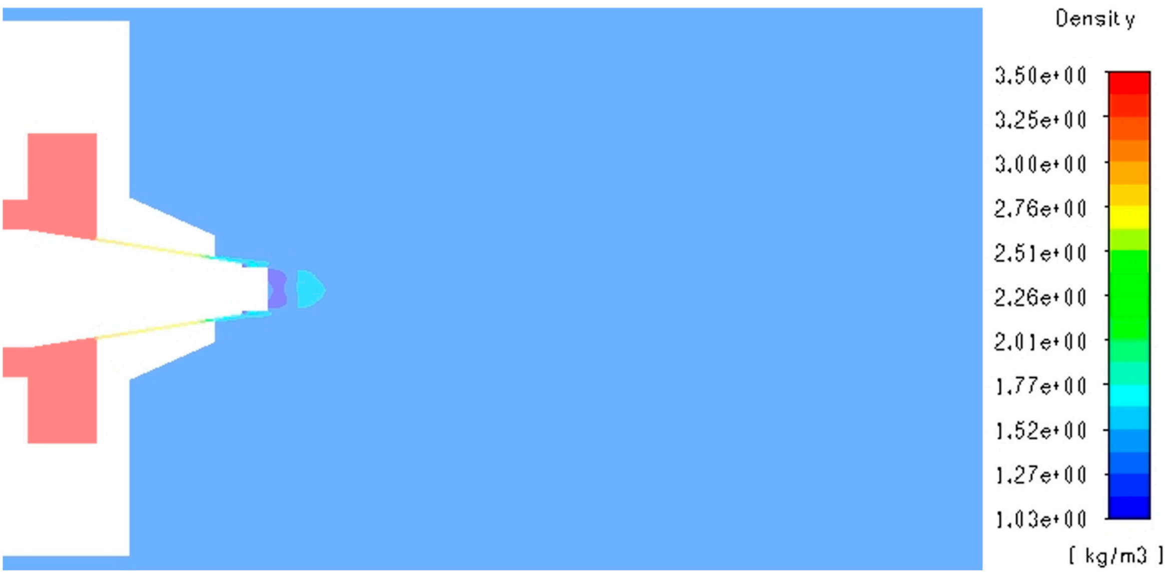

Figure S35. Density contour of k-ε turbulent flow through SBS nozzle at 2 bar.

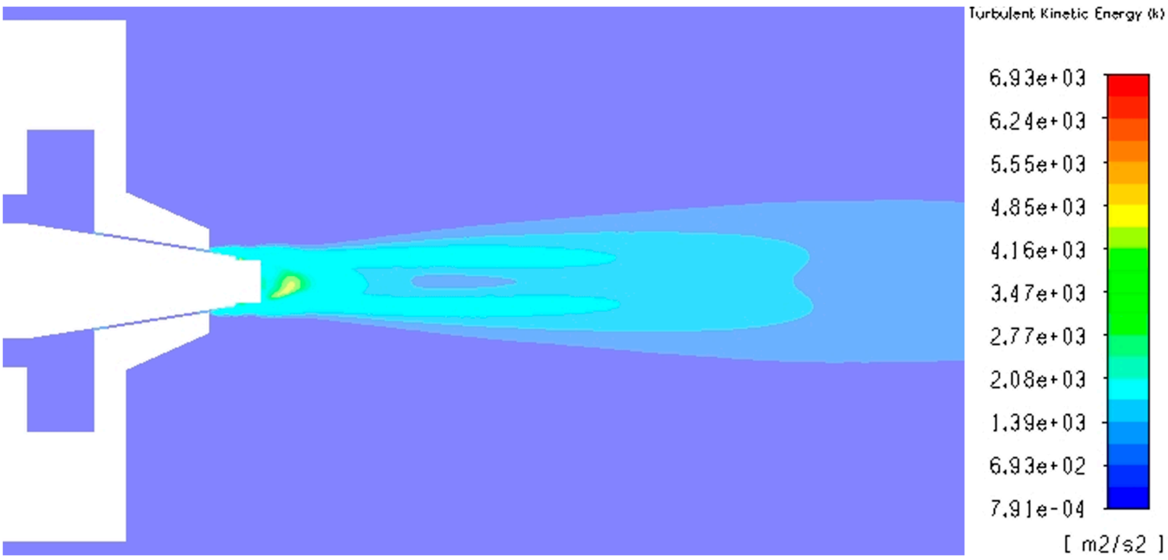

Figure S36. Turbulent kinetic energy (J/kg = m^2/s^2) contour of k-ε turbulent flow through SBS nozzle at 2 bar.

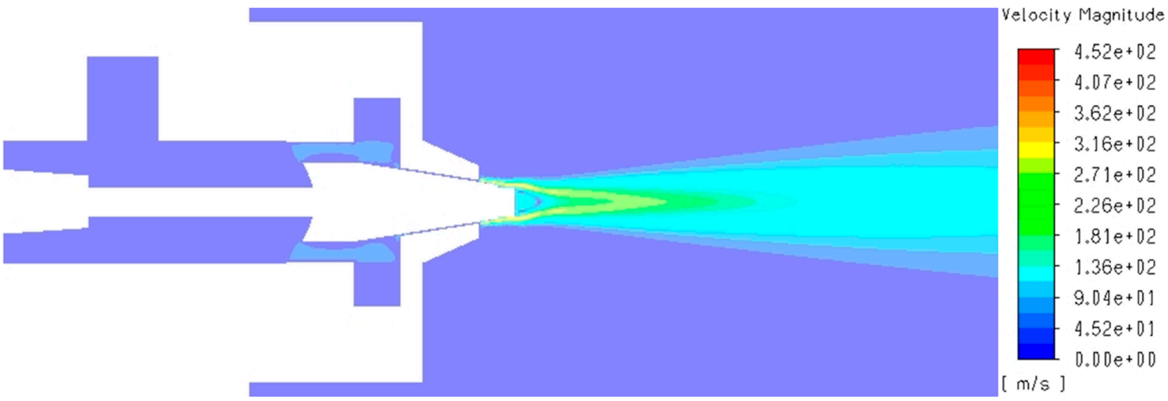

Figure S37. Velocity contour of k-ε turbulent flow through SBS nozzle at 3 bar.

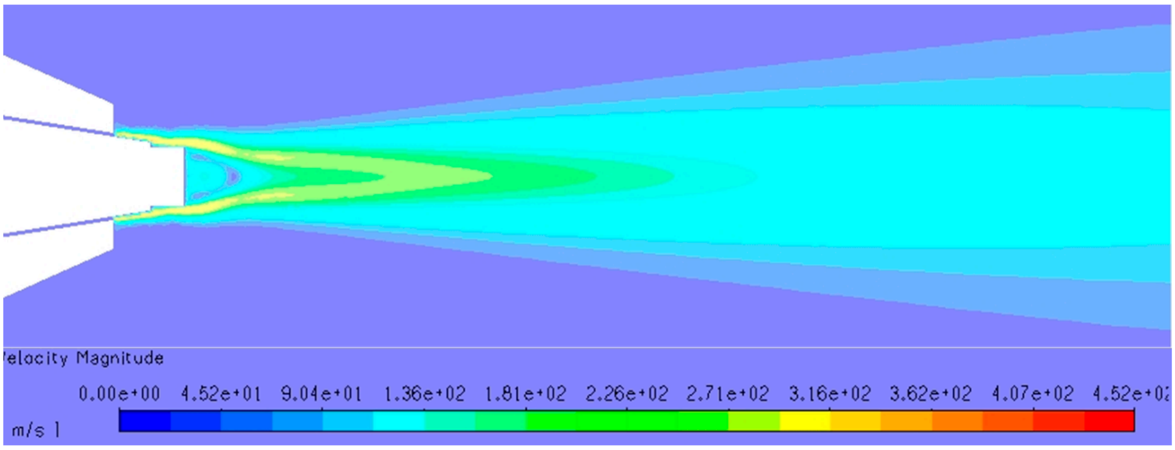

Figure S38. Velocity contour of k-ε turbulent flow through SBS nozzle at 3 bar.

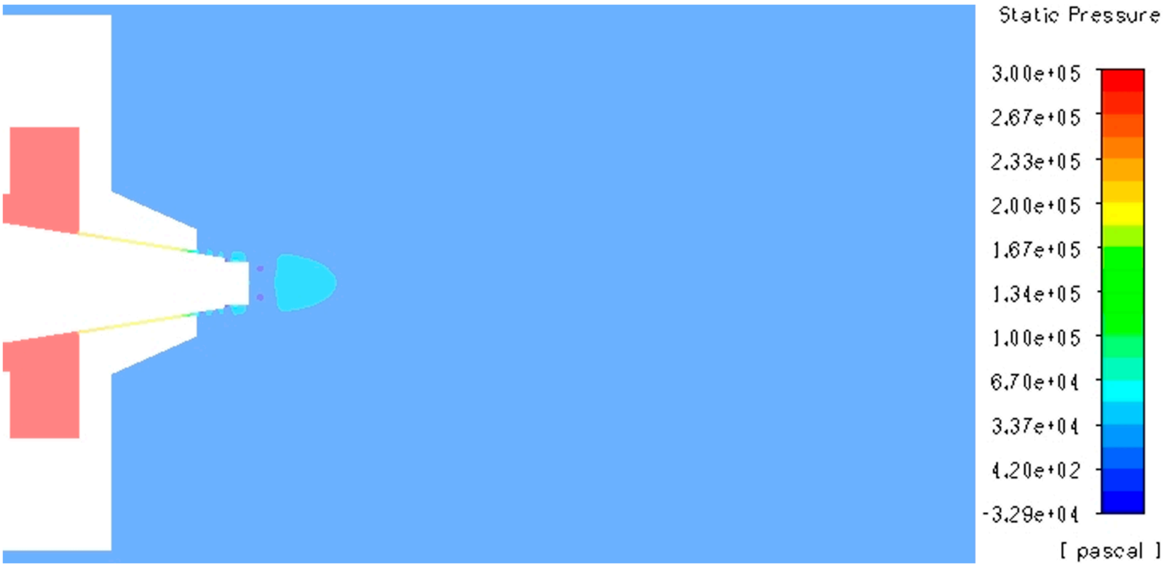

Figure S39. Pressure contour of k-ε turbulent flow through SBS nozzle at 3 bar.

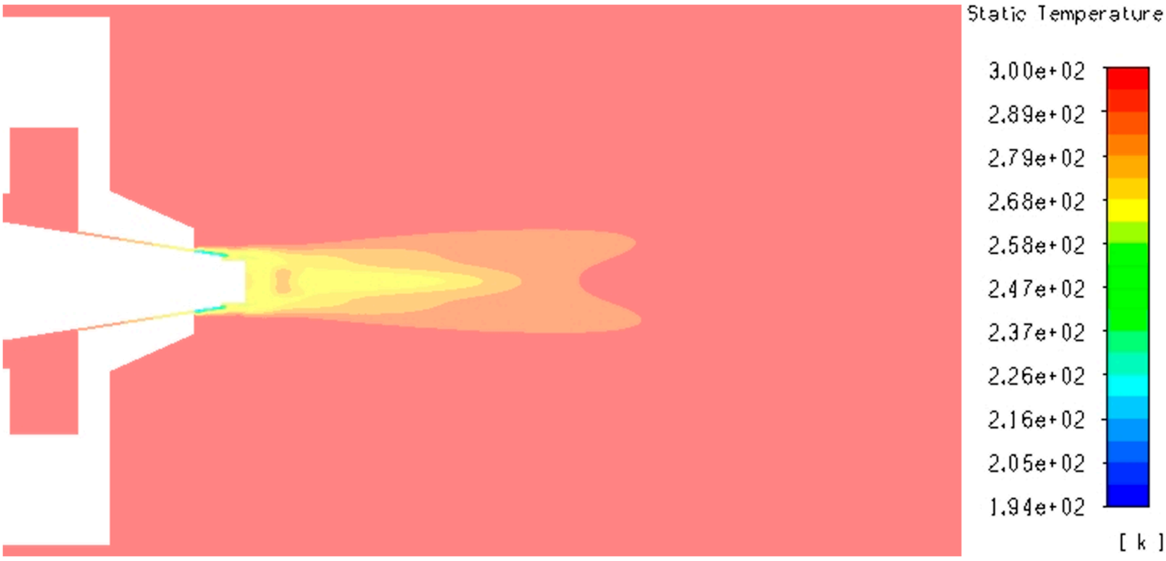

Figure S40. Temperature contour of k-ε turbulent flow through SBS nozzle at 3 bar.

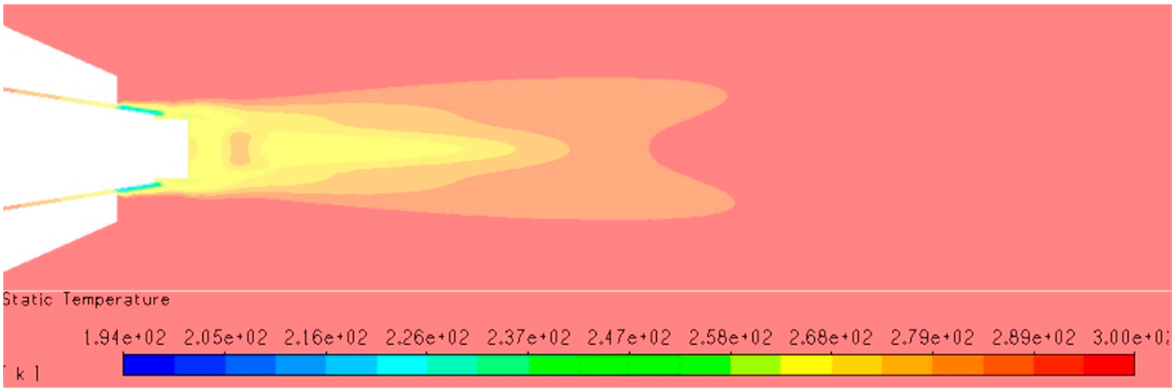

Figure S41. Temperature contour of k-ε turbulent flow through SBS nozzle at 3 bar.

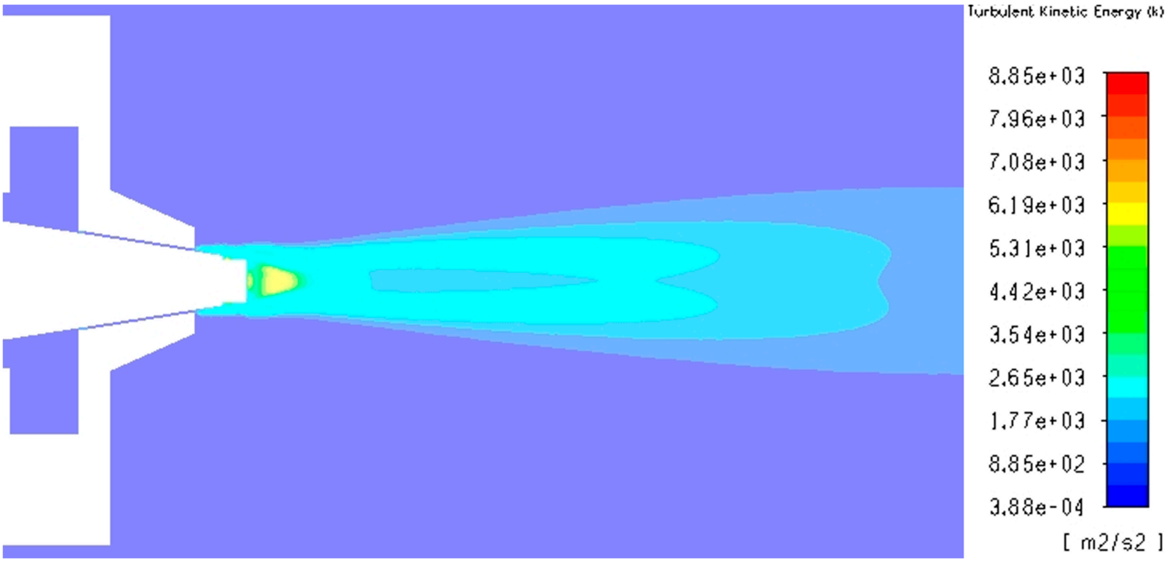

Figure S42. Turbulent kinetic energy (J/kg = m^2/s^2) contour of k-ε turbulent flow through SBS nozzle at 3 bar.

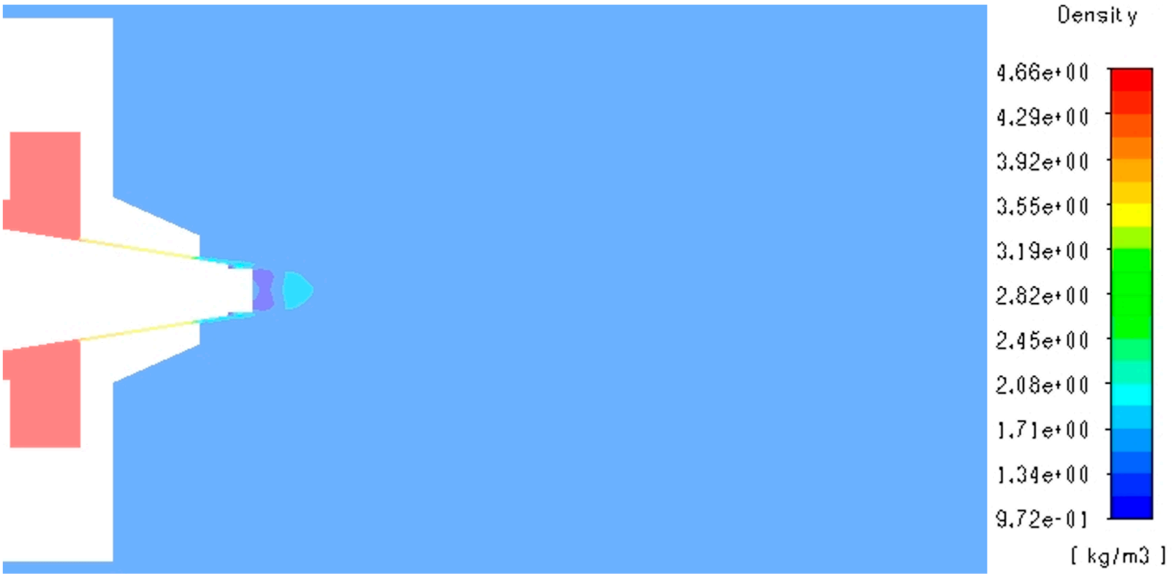

Figure S43. Density contour of k-ε turbulent flow through SBS nozzle at 3 bar.

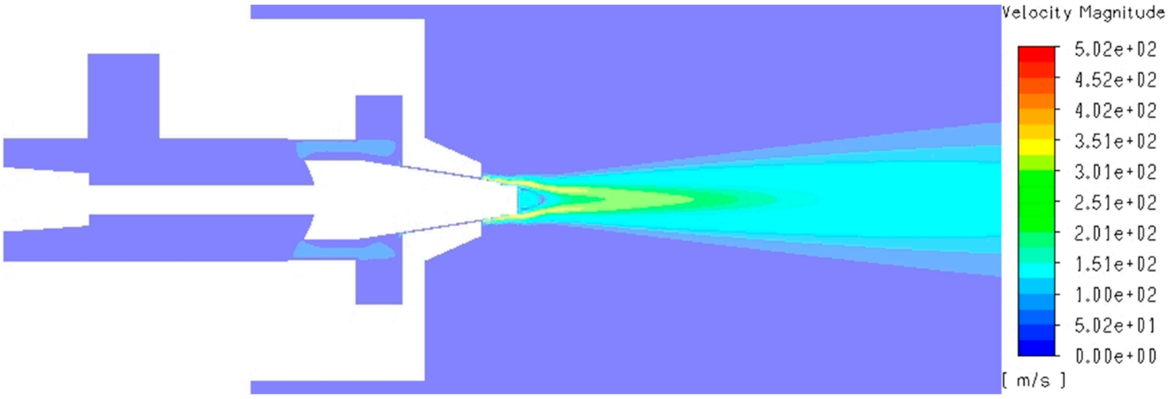

Figure S44. Velocity contour of k-ε turbulent flow through SBS nozzle at 4 bar.

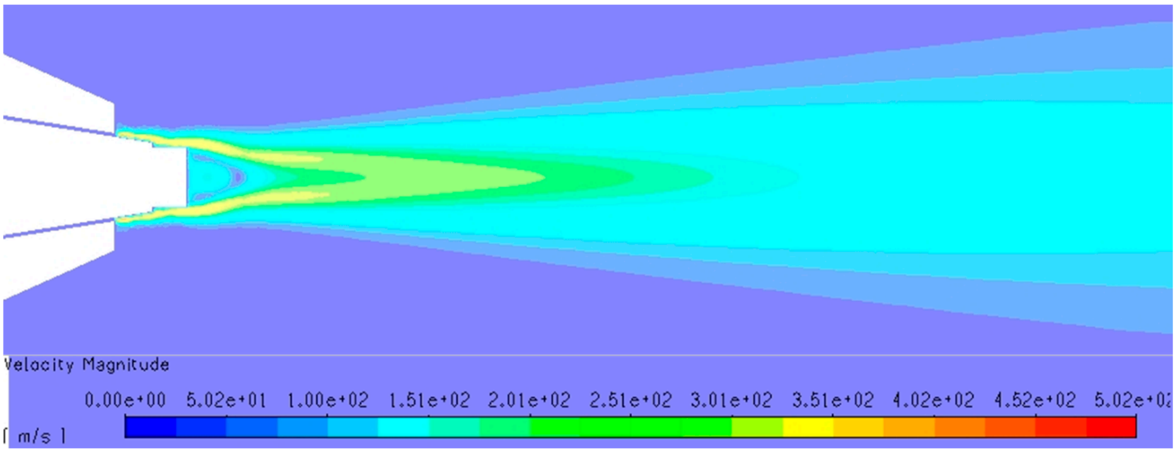

Figure S45. Velocity contour of k-ε turbulent flow through SBS nozzle at 4 bar.

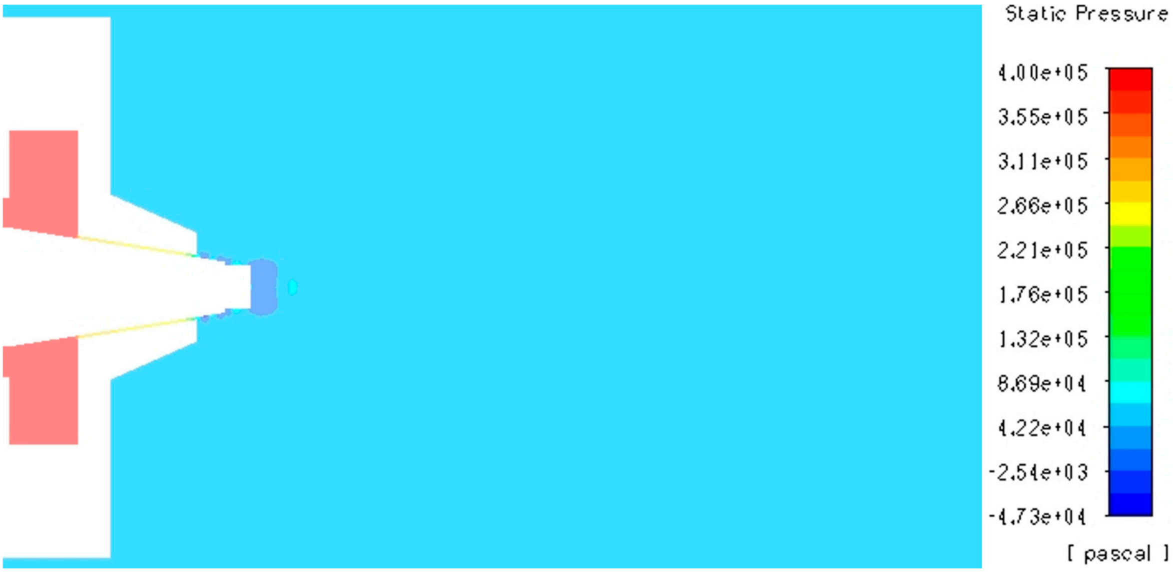

Figure S46. Pressure contour of k-ε turbulent flow through SBS nozzle at 4 bar.

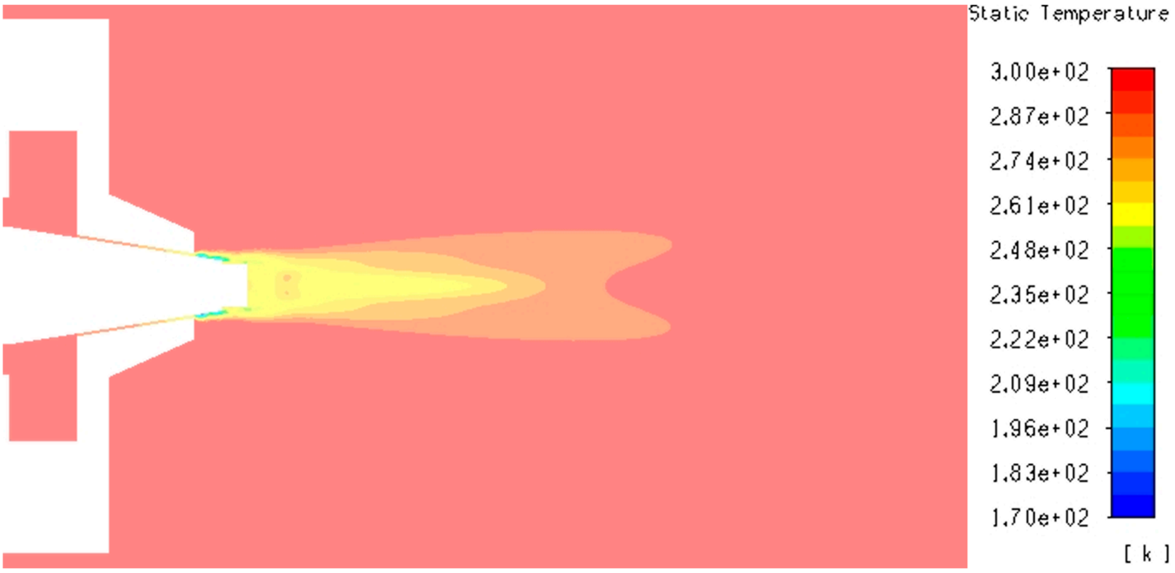

Figure S47. Temperature contour of k-ε turbulent flow through SBS nozzle at 4 bar.

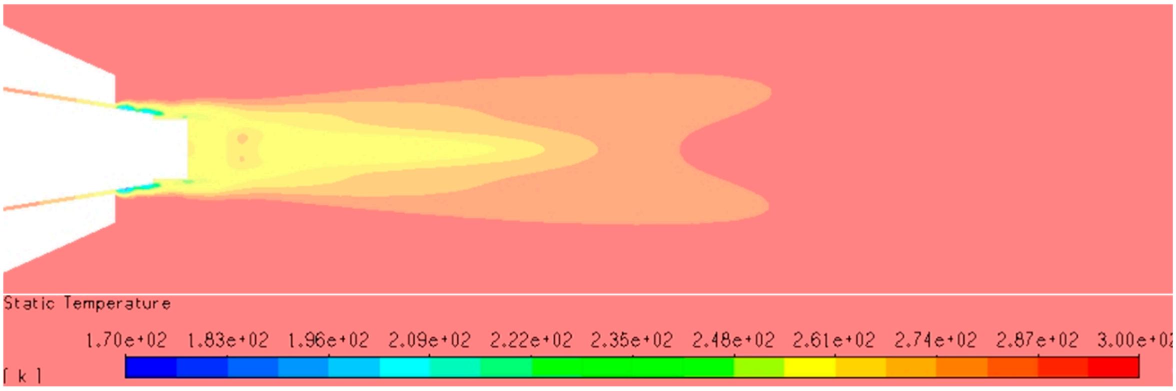

Figure S48. Temperature contour of k-ε turbulent flow through SBS nozzle at 4 bar.

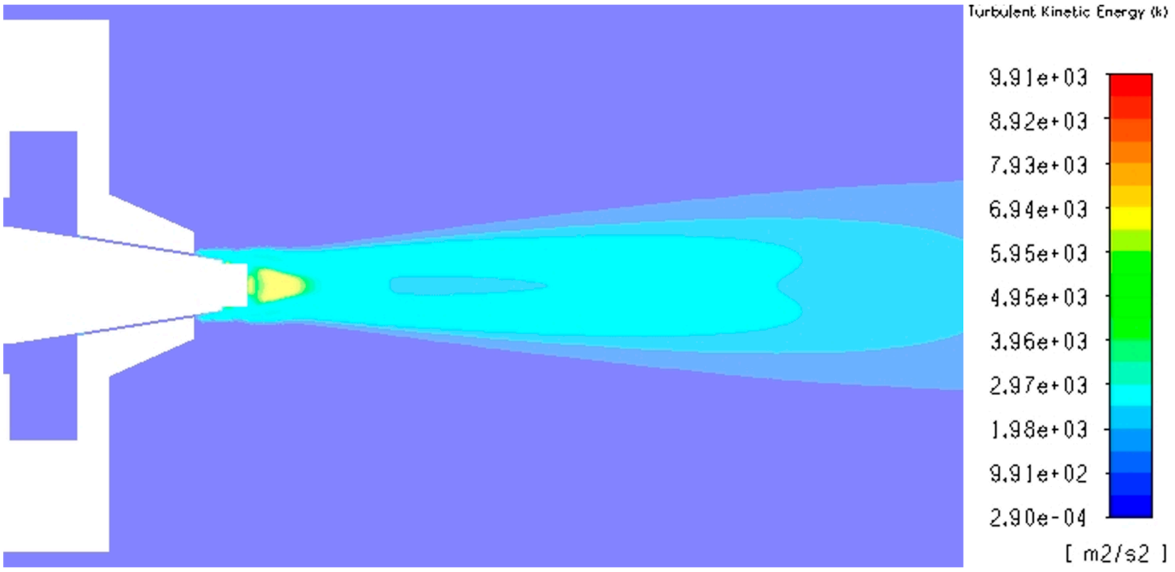

Figure S49. Turbulent kinetic energy contour of k-ε turbulent flow through SBS nozzle at 4 bar.

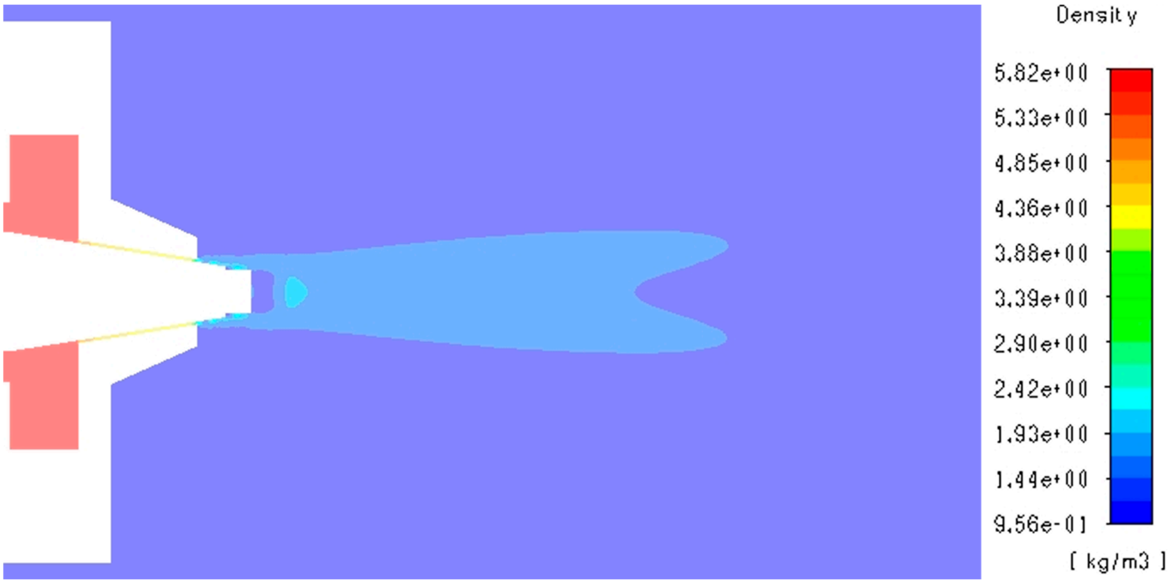

Figure S50. Density contour of k-ε turbulent flow through SBS nozzle at 4 bar.

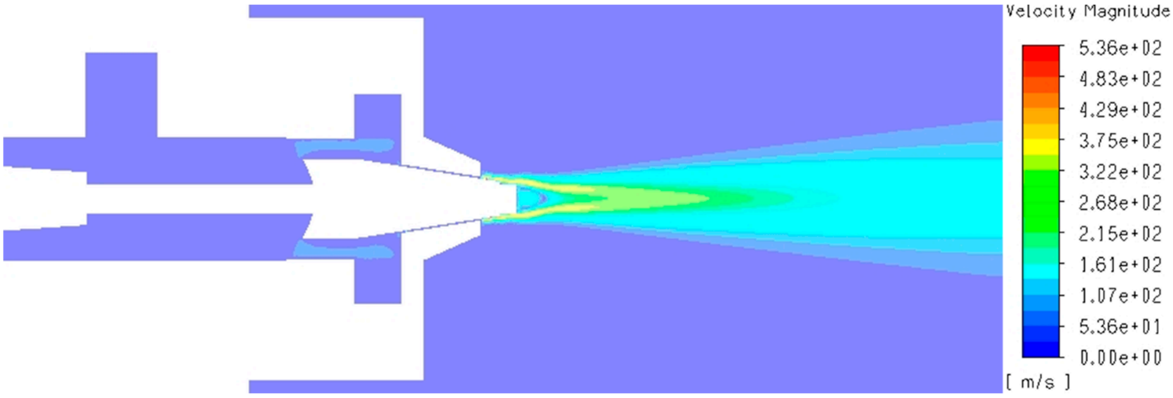

Figure S51. Velocity contour of k-ε turbulent flow through SBS nozzle at 5 bar.

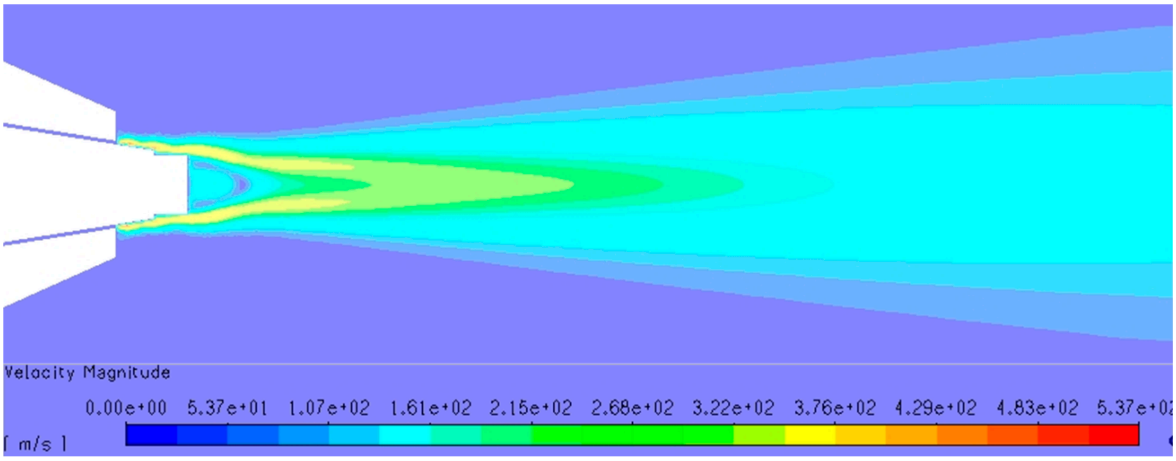

Figure S52. Velocity contour of k-ε turbulent flow through SBS nozzle at 5 bar.

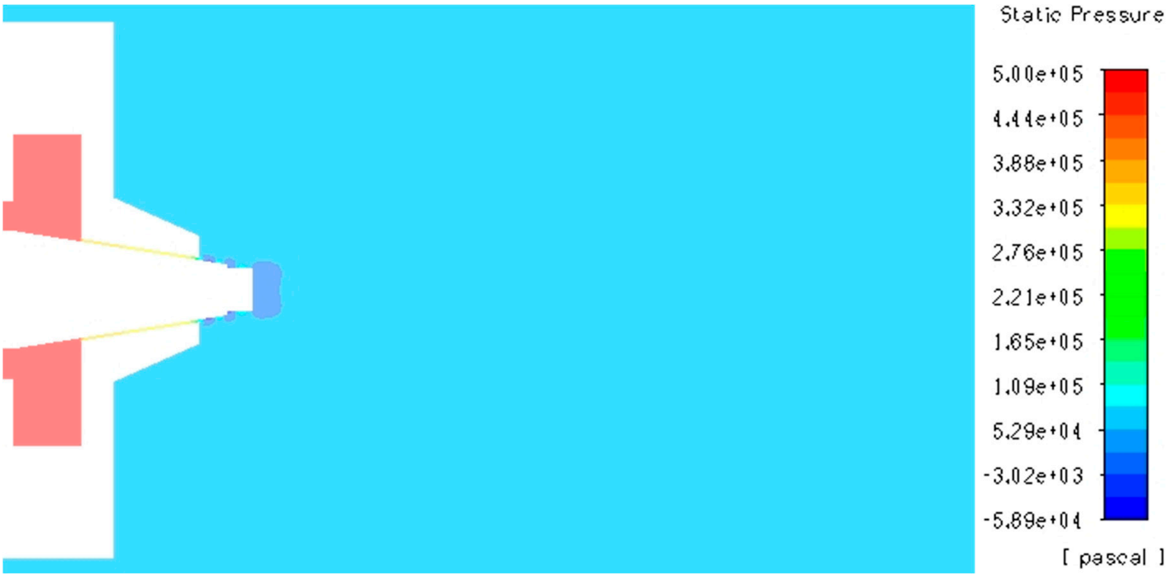

Figure S53. Pressure contour of k-ε turbulent flow through SBS nozzle at 5 bar.

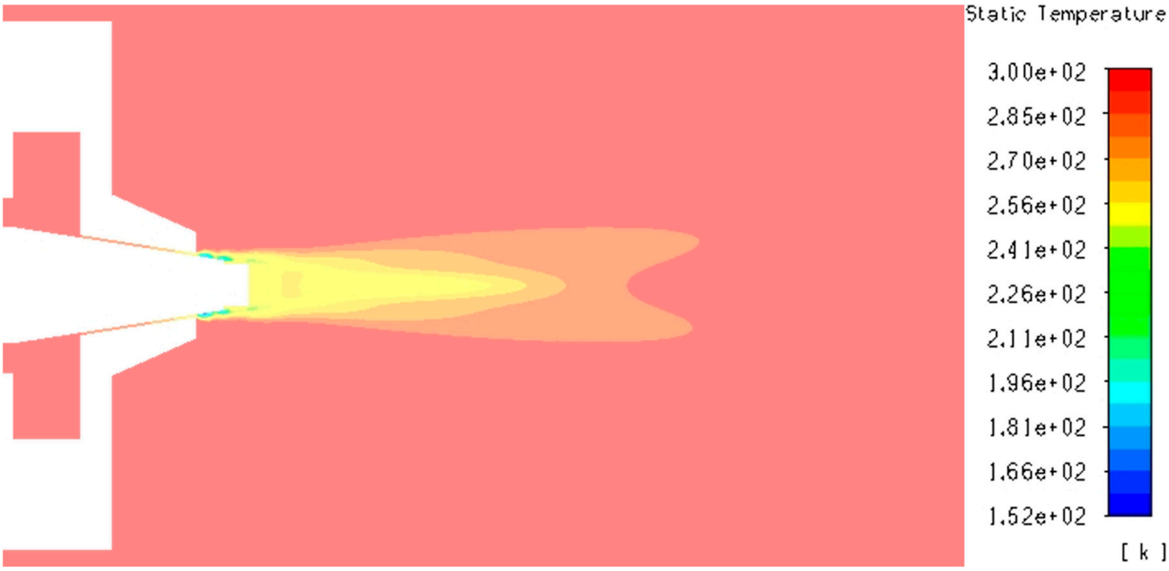

Figure S54. Temperature contour of k-ε turbulent flow through SBS nozzle at 5 bar.

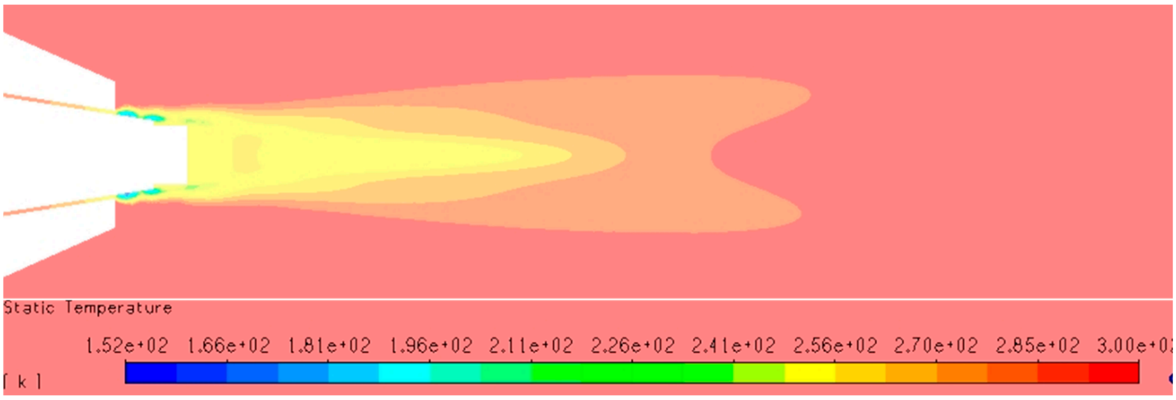

Figure S55. Temperature contour of k-ε turbulent flow through SBS nozzle at 5 bar.

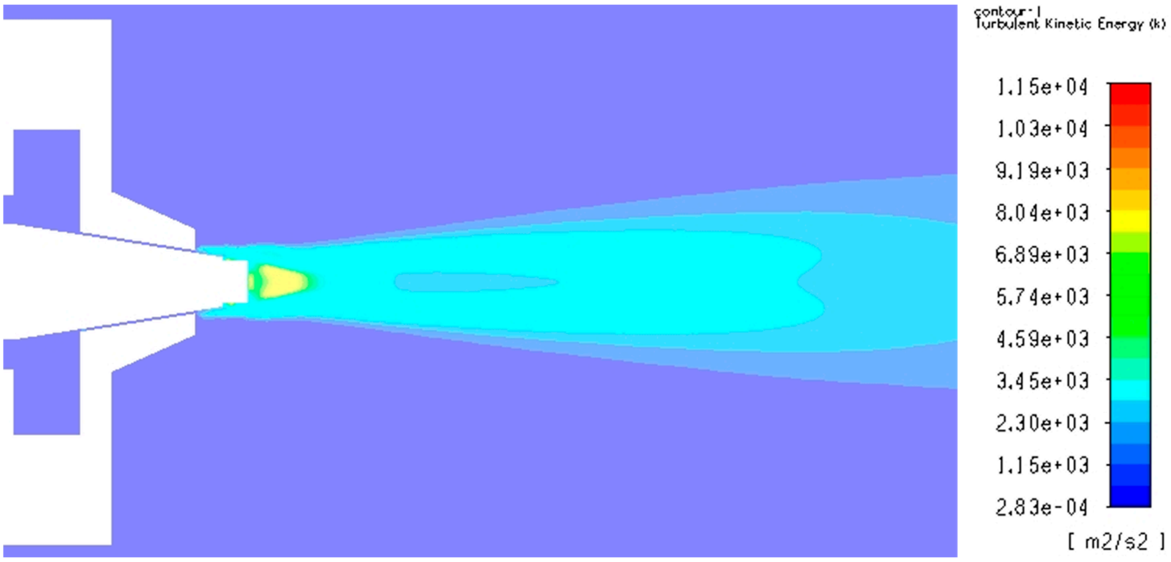

Figure S56. Turbulent kinetic energy contour of k-ε turbulent flow through SBS nozzle at 5 bar.

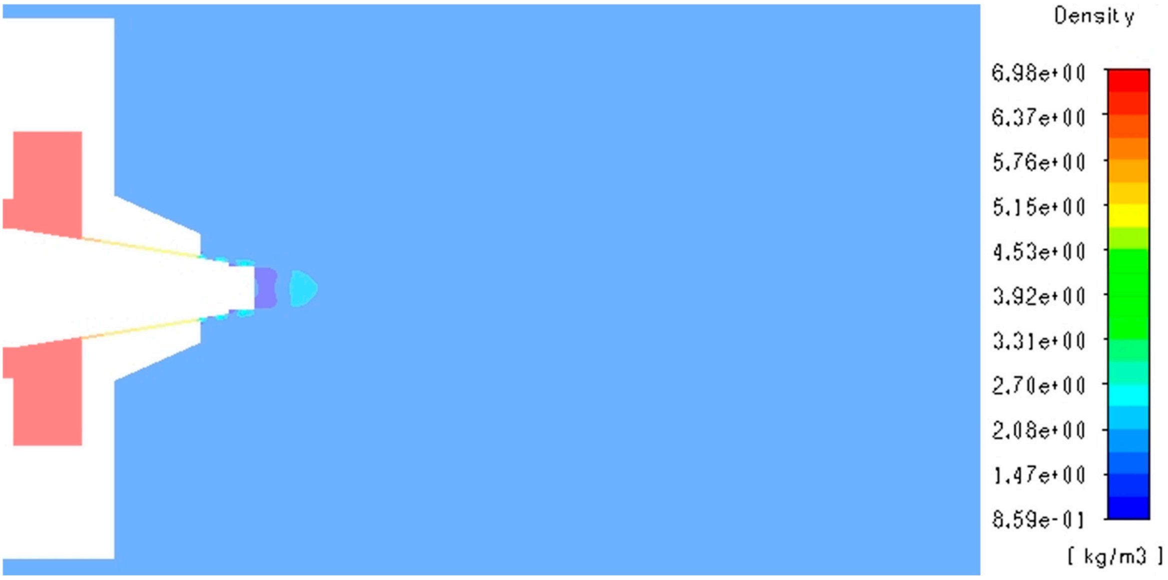

Figure S57. Density contour of k-ε turbulent flow through SBS nozzle at 5 bar.

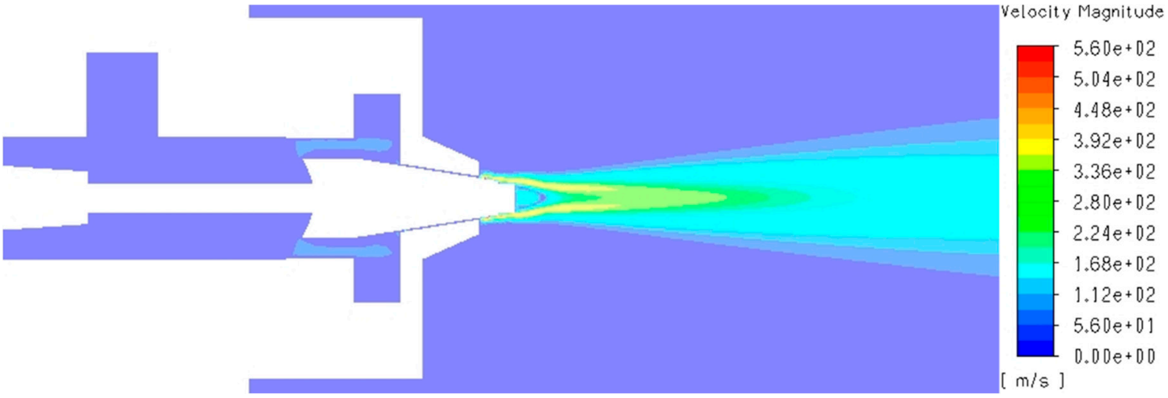

Figure S58. Velocity contour of k-ε turbulent flow through SBS nozzle at 6 bar.

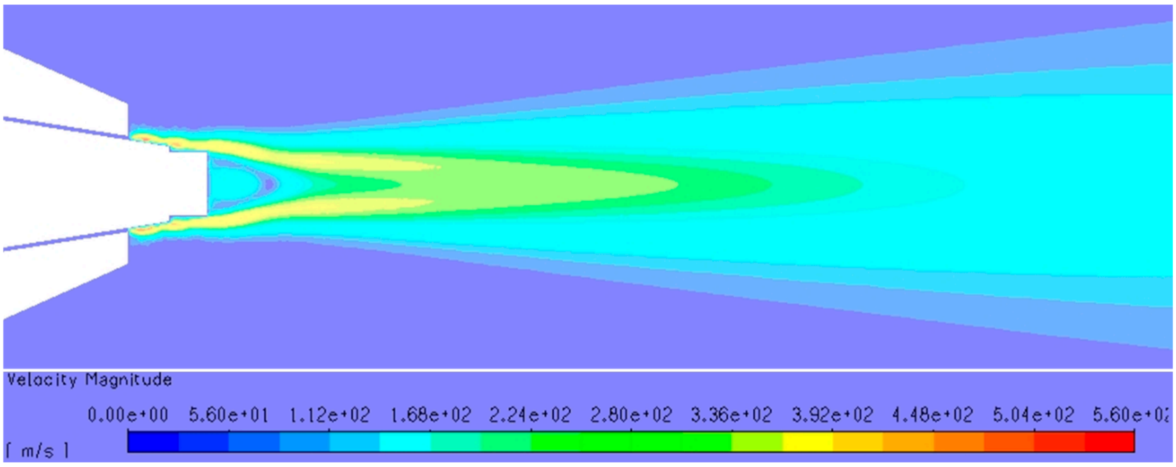

Figure S59. Velocity contour of k-ε turbulent flow through SBS nozzle at 6 bar.

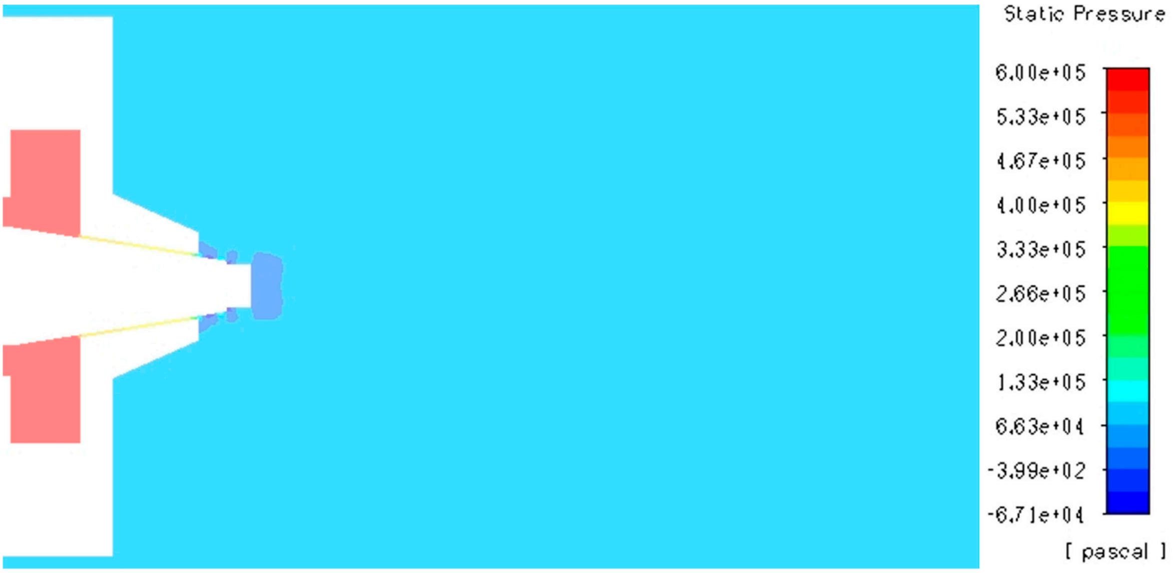

Figure S60. Pressure contour of k-ε turbulent flow through SBS nozzle at 6 bar.

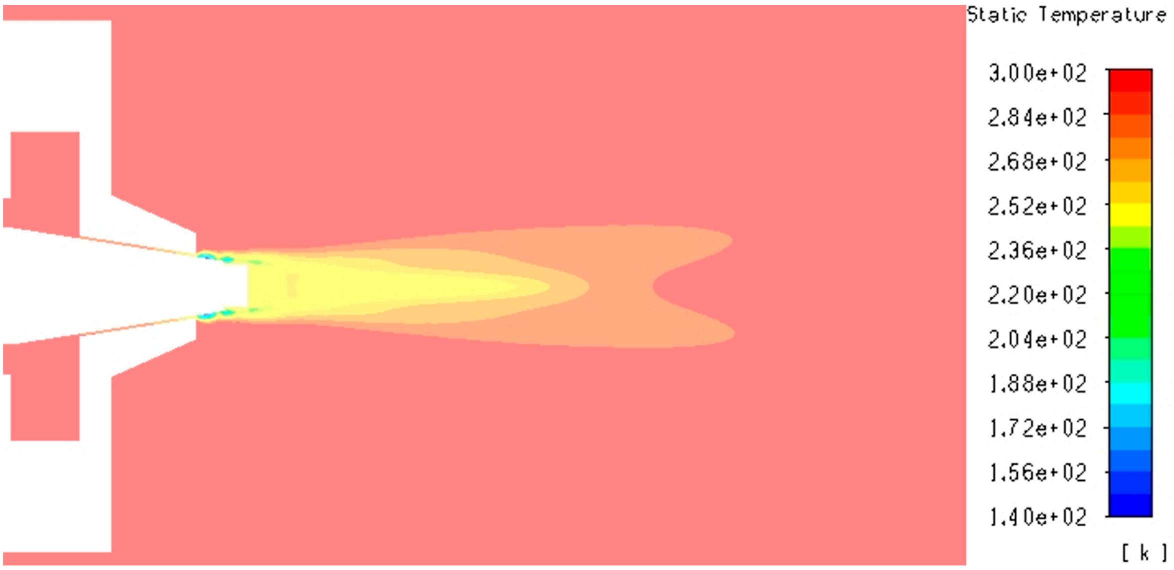

Figure S61. Temperature contour of k-ε turbulent flow through SBS nozzle at 6 bar.

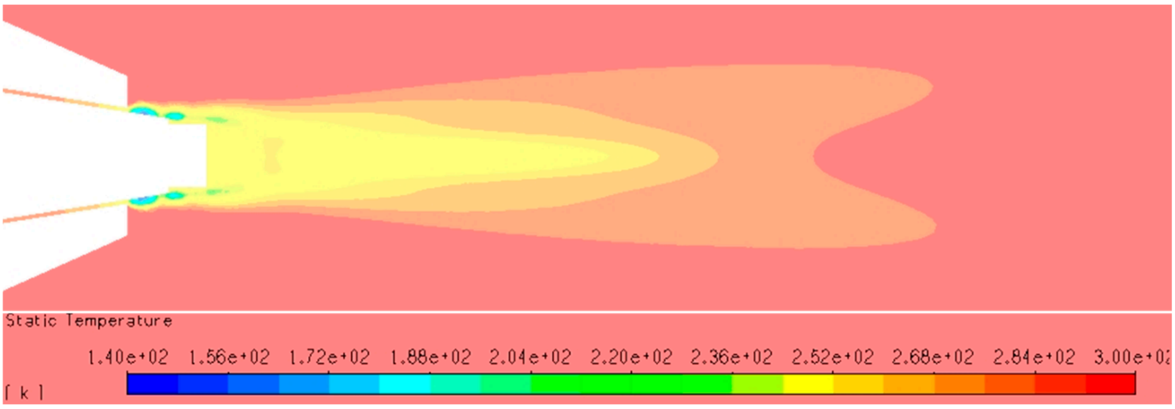

Figure S62. Temperature contour of k-ε turbulent flow through SBS nozzle at 6 bar.

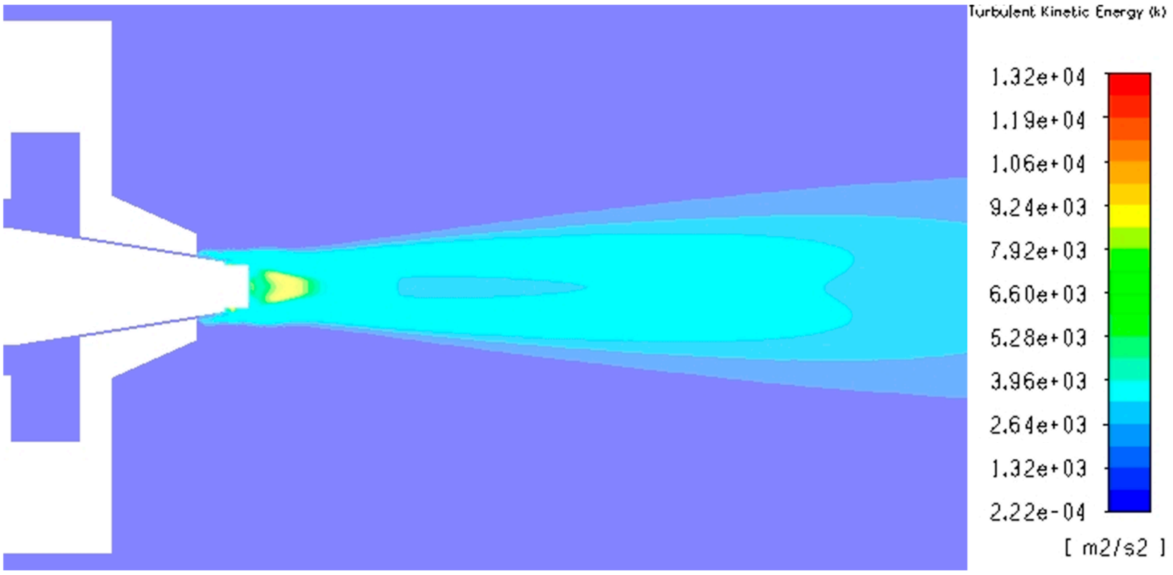

Figure S63. Turbulent kinetic energy contour of k-ε turbulent flow through SBS nozzle at 6 bar.

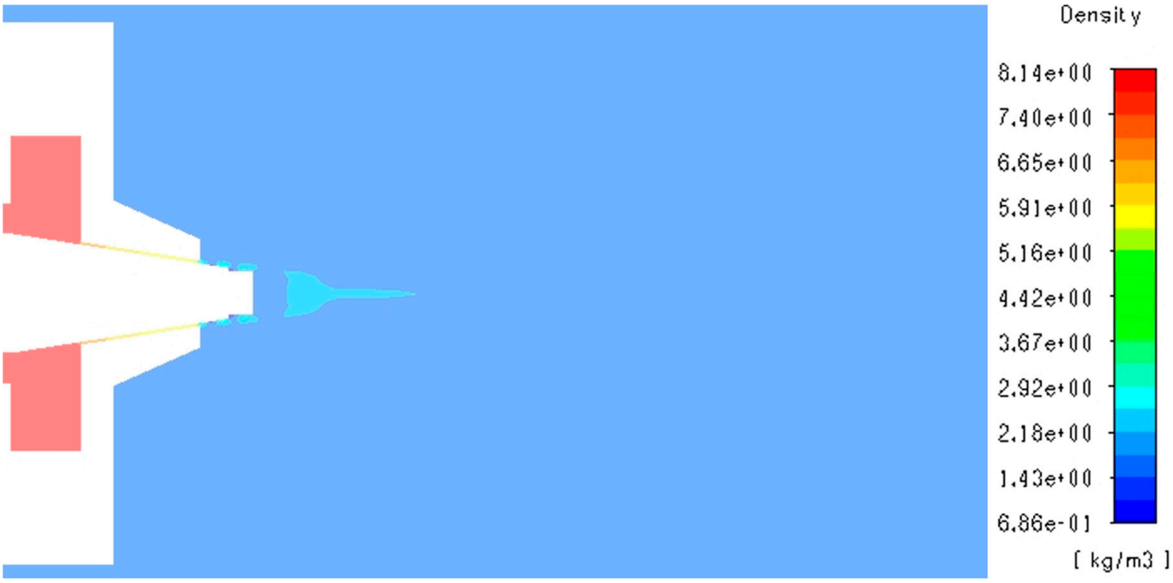

Figure S64. Density contour of k-ε turbulent flow through SBS nozzle at 6 bar.

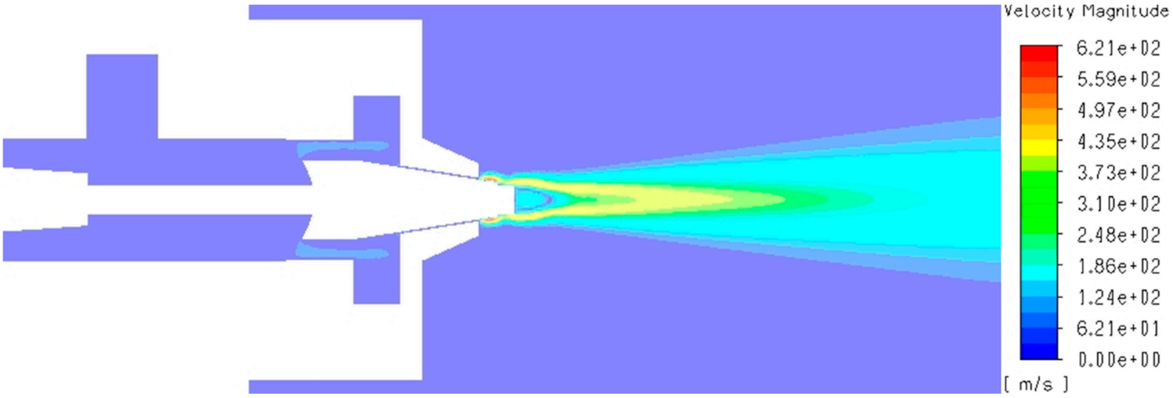

Figure S65. Velocity contour of k-ε turbulent flow through SBS nozzle at 10 bar.

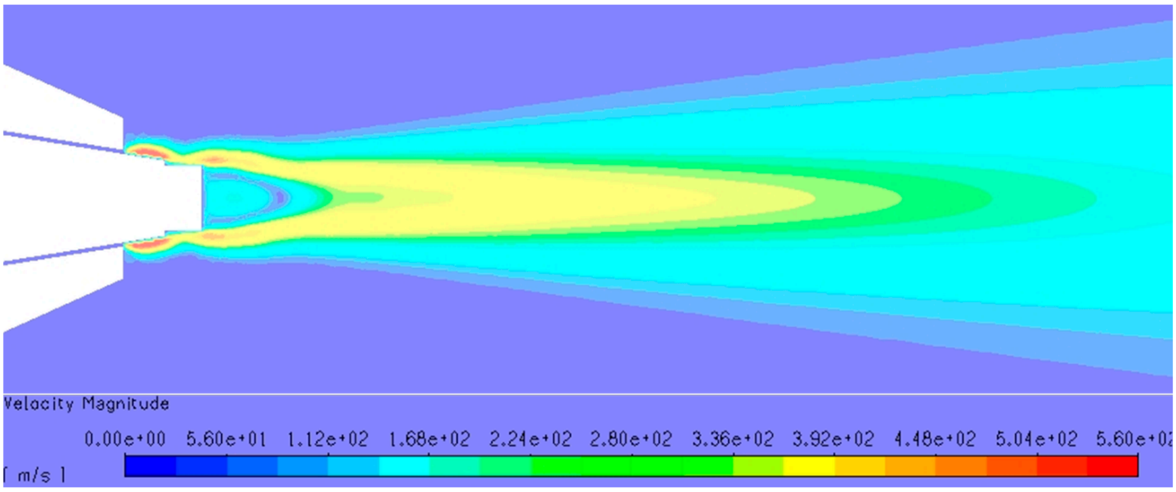

Figure S66. Velocity contour of k-ε turbulent flow through SBS nozzle at 10 bar.

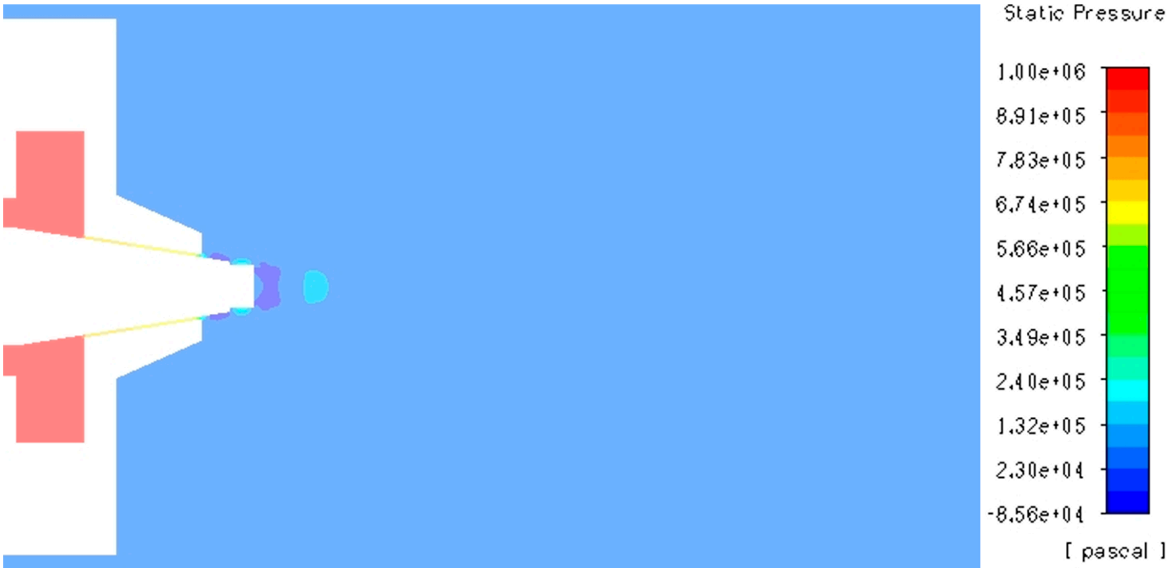

Figure S67. Pressure contour of k-ε turbulent flow through SBS nozzle at 10 bar.

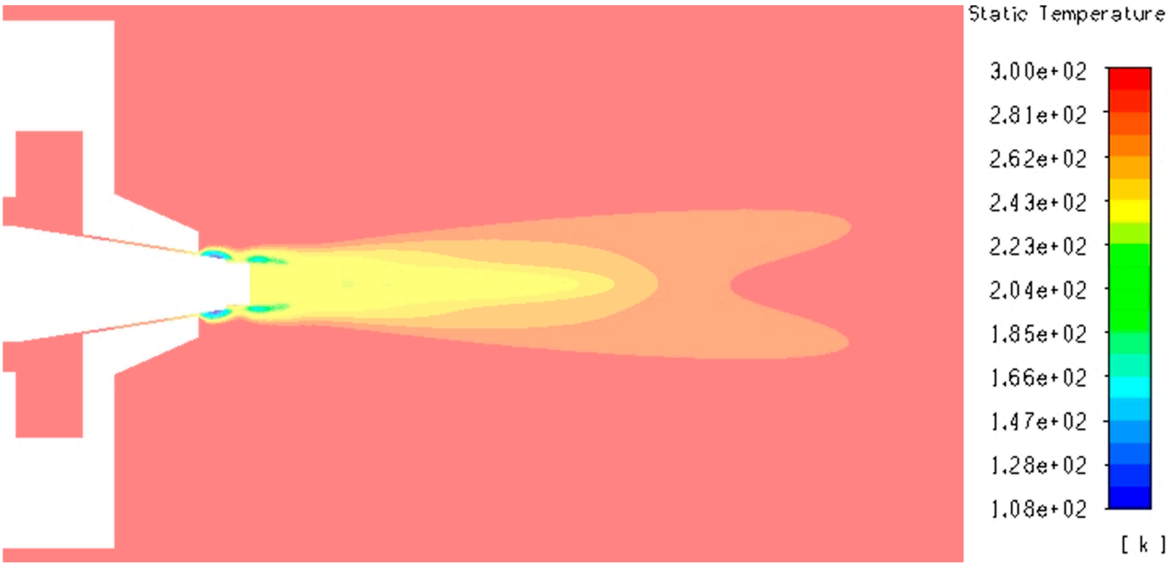

Figure S68. Temperature contour of k-ε turbulent flow through SBS nozzle at 10 bar.

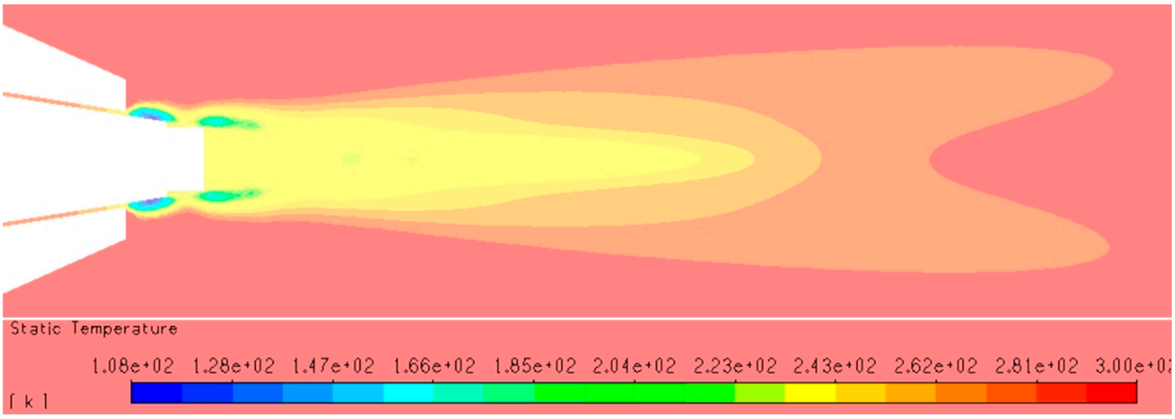

Figure S69. Temperature contour of k-ε turbulent flow through SBS nozzle at 10 bar.

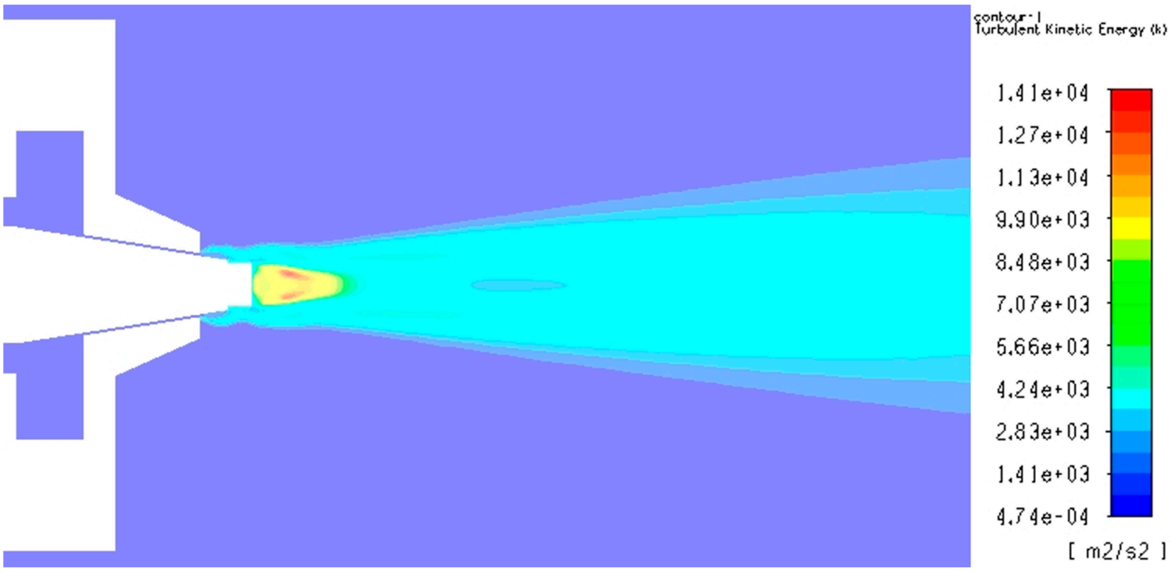

Figure S70. Turbulent kinetic energy contour of k-ε turbulent flow through SBS nozzle at 10 bar.

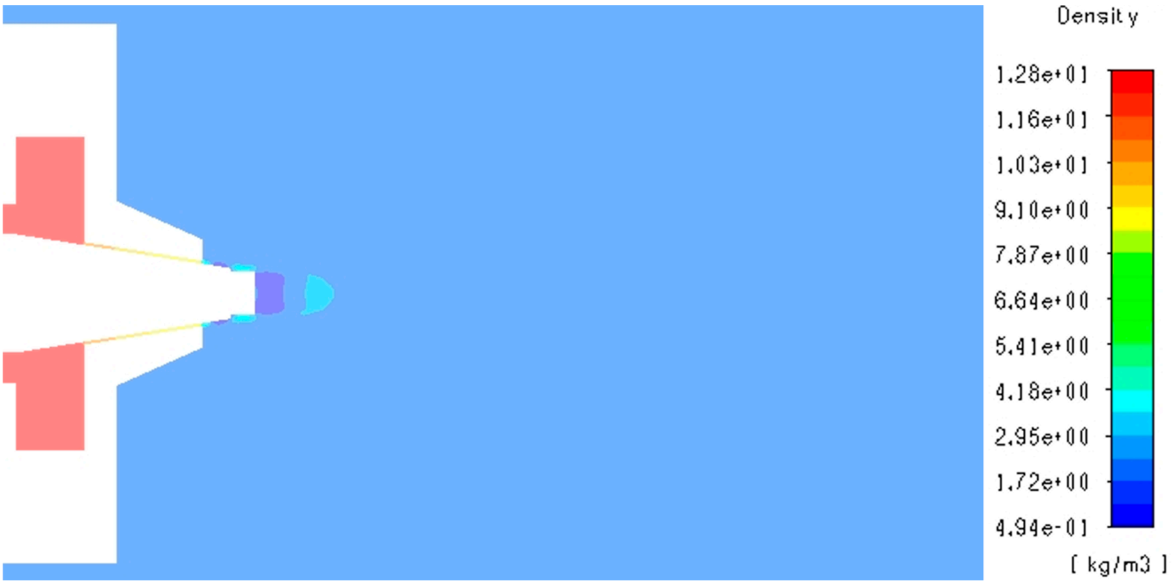

Figure S71. Density contour of k-ε turbulent flow through SBS nozzle at 10 bar.

4. Acknowledgements:

The authors would like to thank British Council for providing funding for the Institutional Links Project (Project ID: 352360451) between Alexandria University, Egypt, and Northumbria University, Newcastle upon Tyne, UK. This work is part of the project of (Newton-Mosharafa Call between UK and Egypt, ID: 30886) which has been funded by Science, Technology & Innovation Funding Authority (STIFA), Egypt.

5. References:

[1] https://www.oxfordreference.com/view/10.1093/oi/authority.20110803100025554.
